# The performance relationship between the EQ-5D-5L composite “Anxiety/Depression” dimension and anxiety and depression symptoms in a large, general population sample

**DOI:** 10.1007/s11136-024-03754-5

**Published:** 2024-09-13

**Authors:** Emily Stella Scott, Erica I. Lubetkin, Mathieu F. Janssen, John N. Yfantopolous, Gouke J. Bonsel, Juanita A. Haagsma

**Affiliations:** 1 Department of Public Health, Erasmus MC, Rotterdam, The Netherlands; 2 Department of Community Health and Social Medicine, CUNY School of Medicine, New York City, NY USA; 3 Section Medical Psychology and Psychotherapy, Department of Psychiatry, Erasmus MC, Rotterdam, The Netherlands; 4 Health Department of Economics, National and Kapodistrian University of Athens, Athens, Greece; 5 EuroQol Research Foundation, Rotterdam, The Netherlands

**Keywords:** Anxiety, Depression, Discriminatory performance, Psychometric properties, Differential item functioning, Optimal cut-off

## Abstract

**Purpose:**

This cross-sectional study aims to understand the relationship between responses on the Anxiety/Depression (A/D) dimension of the EQ-5D-5L and symptoms of anxiety and depression on the GAD-7 and PHQ-9 instruments. In doing so, we investigate the comparative performance of the dimension between diagnostic groups (i.e. anxiety (GAD-7); depression (PHQ-9); anxiety & depression versus none). We additionally investigate the discriminatory performance between sub-populations based on gender, age, education and self-reported chronic conditions.

**Methods:**

19,902 general population participants completed a health survey in May/June 2020, from five European countries and the United States. Performance of A/D was calculated using the Area Under the Receiver Operating Characteristic curve (AUROC), and was compared to having anxiety (GAD-7 ≥ 8), depression (PHQ-9 ≥ 10) and both versus none for the total population and sub-populations. Several additional sensitivity analyses were conducted, including calculations of the optimal A/D cut-off.

**Results:**

The performance in the total sample was good (AUROC > 0.8) and did not differ significantly between diagnostic groups. The performance differed significantly between the age groups, with worse performance in the younger groups, and differed between those with a singular chronic condition, with worse performance in those indicating having an anxiety or depression disorder. The performance did not differ significantly by gender, education, nor total chronic conditions.

**Conclusion:**

The A/D dimension captures symptoms of anxiety, depression or both equally well. Performance is worse in the younger population. Interpretation in those with a self-reported anxiety or depression disorder should be further investigated. This is the first-of-its-kind large population sample performance analysis, where we present evidence that the performance of the A/D dimension differs between ages, and thus intra-age comparative results may be flawed.

**Supplementary Information:**

The online version contains supplementary material available at 10.1007/s11136-024-03754-5.

## Introduction

Health-related quality of life (HRQOL) is a subjective, multi-dimensional concept that constitutes physical and social functioning, pain and psychological symptoms, and more [1]. The EQ-5D-5L is a generic instrument measuring HRQOL in five short questions referring to “today” [2, 3]. These include core aspects of HRQOL that are summarised in dimensions (5D): Mobility, Self-care, Usual Activities, Pain/Discomfort and Anxiety/Depression. The EQ-5D-5L is widely applied in clinical, population and economic studies [3, 4], informing patient management and policy decisions [5]. Given its broad application, it is important the instrument is concise and easy to use, yet an accurate metric and reliable for use in (sub-)populations [5].

The Anxiety/Depression dimension (A/D) of the EQ-5D-5L covers psychological symptoms within HRQOL, as demonstrated using confirmatory factor analysis [6]. The dimension has a composite formation, because it consists of two distinct nosological concepts – anxiety and depression. The formation requires the respondent to provide a single response on the level of severity of these two symptoms. Yet how it is interpreted and answered by respondents is multifactorial and complex. The anxiety and depression terms were chosen because anxiety and depressive disorders are the most prevalent specific psychological conditions departing from the healthy state [7], and commonly are co-morbid conditions in general and clinical populations [8].

Instruments are required to be adequately reliable and valid in order to provide legitimately useful and meaningful results. As the A/D dimension covers psychological symptoms, it is required that the dimension adequately captures anxiety and depression symptoms. Using the Area Under the Receiver Operating Characteristic curve (AUROC), which quantifies the overall ability of a test to discriminate between two groups by integrating sensitivity and specificity into a performance value, one can determine whether the same underlying construct is measured between two instruments [9]. In previous studies that analysed the performance of the A/D dimension as a screening tool for psychiatric conditions, it was found to perform fairly well (AUROC = 0.78–0.86) in screening a community population (90 days post-discharge from hospital), poorly (AUROC = 0.70–0.74) in a hospital population [10], and well in a diabetic adult population (AUROC = 0.88–0.92) [11]. The discriminatory performance of the A/D dimension for detection of anxiety or depression symptoms equalled that of in-depth screening instruments for anxiety and depression.

As the above studies illustrate, the A/D dimension is used in a wide variety of populations given the EQ-5D’s broad applications, meaning it must consistently discriminate across different populations. Thus, it is essential to understand the relationship between responses on the A/D dimension and symptoms of anxiety and depression for informing on the EQ-5D-5L A/D dimension’s validity in different populations, to ensure that comparisons across groups are valid and meaningful. Therefore, the objective is to estimate the discriminatory performance of the composite A/D dimension of the EQ-5D-5L instrument in capturing anxiety and depression symptoms as measured by the Generalised Anxiety Disorder (GAD-7) instrument and the Patient Health Questionnaire (PHQ-9) instrument, respectively. Additionally, we explored the performance of the A/D dimension between sub-populations of a general population sample based on differences by gender, age, education level and chronic conditions.

## Methods

### Population, data collection and consent

Participant data for this secondary analysis were used from the POPulation health impact of the CORoNavirus disease 2019 (COVID-19) pandemic (POPCORN) study, a longitudinal study that aimed to investigate the broader effects of the COVID-19 pandemic on HRQOL and mental health of the general population in various countries. Participants were enlisted by a market research agency to which written informed consent was provided upon registration to the agency’s voluntary panels. Upon enlisting, the general population participants were aged 18–75. The sample was by design representative for age, sex and education within each country. Data was collected via web-based surveys that were first distributed in early 2020, and then annually until 2023. Once a participant started the survey, the data collection system would not allow skipping or missing questions. A small reward in the form of cash or points was provided by the agency upon completion. The data were anonymised by the agency.

This methodological study is analysing data collected April-May 2020, from six countries: Greece, Italy, The Netherlands, Sweden, the United Kingdom (UK) and the United States (US). For the EQ-5D questionnaires all official EQ-5D-5L translations were used. Where available, official translations for the GAD-7 and PHQ-9 were used; this did not include Swedish. For the remaining questions, the surveys were translated into the respective national language of the country by the agency. The translations were cross-checked by bilingual speakers who had a scientific background. There are no missing values as the survey system does not allow for unanswered or skipped questions.

### Measures of anxiety and depression

The last question of the EQ-5D-5L is the A/D dimension. The instrument refers to a period of “today”. Participants rate the level of severity of their problems on a 5-Level (5 L) scale, as either “none”, “slight”, “moderate”, “severe” or “unable to/extreme”, hence level scores range from 1 to 5, respectively [2].

The GAD-7 is a 7-item questionnaire that aims to detect generalised anxiety and other anxiety disorders [12]. The PHQ-9 is a 9-item questionnaire that aims to detect depressive disorders cf. DSM-IV [13]. Both instruments refer to a period over the last two weeks. The GAD-7 includes questions on symptoms of nervousness, worry, irritability, etc., and the PHQ-9 on symptoms of hopelessness, little energy, and more. Participants rate their symptom frequencies between “0 = not at all” to “3 = nearly every day” on a 4-item ordinal scale. Therefore, the total minimum score is 0 and maximum scores are 21 and 27 for the GAD-7 and PHQ-9, respectively. Based on the total score, the severity is categorised as mild, moderate, moderately severe (only for the PHQ-9) or severe, with cut-offs of 5 and above, 10, 15, and 20 (only PHQ-9), respectively. Based on the literature, we used a cut-off score of ≥ 8 [14, 15] from which to differentiate anxiety from no anxiety and of ≥ 10 from which to differentiate depression [13; 16].

As the GAD-7 and PHQ-9 instruments are specific to measuring anxiety and depression, respectively, using a comprehensive list of symptom-related questions, these instruments were used in our study as the “gold standards” to compare the A/D dimension with. In general population samples, the GAD-7 [17] and PHQ-9 [18] were found to have good construct validity and reliability (internal consistency) (Cronbach’s α = 0.89 & 0.87, respectively).

### Data analysis

The diagnostic groups that were used to evaluate the performance of the A/D dimension were split into anxiety (score of GAD-7 ≥ 8), depression (PHQ-9 ≥ 10) and co-morbid anxiety and depression (GAD-7 ≥ 8 & PHQ-9 ≥ 10). As the cases in these groups are not exclusive (i.e. some cases occur in more than one group), for the descriptive statistics only, four mutually exclusive groups were created, which we call here the diagnostic *sub-*groups. These are defined as:

- No anxiety (GAD-7 < 8) and no depression (PHQ-9 < 10)
- Anxiety present (GAD-7 ≥ 8) and no depression (PHQ-9 < 10)
- No anxiety (GAD-7 < 8) and depression present (PHQ-9 ≥ 10)
- Anxiety (GAD-7 ≥ 8) and depression present (PHQ-9 ≥ 10)

For analysing the descriptive statistics between the diagnostic sub-groups, the chi-square, Fisher’s exact and ANOVA tests were used to test for statistically significant differences in the number of observations between groups.

In order to examine the performance of the A/D dimension, we used the AUROC analysis. The AUROC is interpreted as the average sensitivity value across all possible specificity values, and, therefore, is a measure of the overall discriminatory performance of a test [19]. A requirement for this analysis is that the outcome variable must be binary (disease present versus absent), which is not possible for the “No anxiety and no depression” sub-group. Therefore, the A/D dimension was compared within their diagnostic group to symptoms of anxiety (versus no anxiety), depression (versus no depression) and both combined (versus having neither anxiety nor depression), as measured by the GAD-7 and PHQ-9. In this way, we compared the performance between instruments, and further compared the performances between sub-populations by splitting the population based on age, gender, education and chronic conditions. Age was numeric and categorised into four age groups: 18–30; 31–45; 46–60; 61–75, because these are equally large intervals of 15 years (with the exception of the youngest age group). The mid-point (age 45) is also the median age in our sample. Gender had three possible outcomes: male, female and other. The highest level of achieved education was categorised according to the International Standard of Classification of Education (ISCED) into low (ISCED 0–2), medium (ISCED 3–4) and high (ISCED 5–8). All or no chronic conditions could be selected from the listed options: Asthma; chronic bronchitis; Severe heart disease; Consequences of a stroke; Diabetes; Chronic rheumatoid arthritis; Severe back complaints/arthrosis of the back; Painful/swollen joints of knee or hip due to arthrosis; Cancer; Memory problems due to a neurological disease/dementia; Memory problems due to ageing; Depression or anxiety disorder, including an open box statement (Other chronic complaints). In this paper also the population with a singular chronic condition (as opposed to none or more than one) was separately analysed, comparing those that selected Depression or anxiety disorder versus those that selected any other chronic condition. The discriminatory performance between diagnostic groups and sub-populations is compared using the AUROC, whereby a larger value is considered to have improved discriminatory performance [9]. The following AUROC value criteria were used: ≤0.5 = useless test; 0.5< - ≤0.7 = poor test; 0.7< - ≤0.8 = moderately accurate test; 0.8< - ≤0.9 = good test; 0.9< - ≤0.99 = excellent test and 1 = perfect test [20]. The AUROC scores were calculated using the parametric method (smoothing) as is recommended for discrete rating scales and large sample sizes [19], as well as their 95% confidence intervals using 2,000 stratified bootstrap repetitions. We additionally conducted sensitivity analyses for all AUROC analyses using the non-parametric method, as the data on the EQ-5D A/D dimensions was not normally distributed [9], as well as using higher thresholds of ≥ 10 and ≥ 15 for the GAD-7 and PHQ-9, respectively. Statistically significant comparisons were made examining solely the AUROC confidence intervals.

To support the performance analysis, we calculated the sensitivity, specificity, Positive Predictive Value (PPV)/Precision, Negative Predictive Value (NPV) and accuracy using different thresholds of the A/D dimension with the diagnostic groups in the total population and by sub-population. To determine the optimal cut-off points for the A/D dimension in the total sample and by sub-population, we calculated the Youden index from the sensitivity and specificity scores [21]. Statistical analyses were carried out using IBM SPSS version 28.0.1.0. For the AUROC analyses we used R Studio Version 4.2.1 and the pROC open-source package [22]. Figures were created using Microsoft Excel.

## Results

### Respondent characteristics and their mental health

In total, 19,902 participants were included in the study. The median age was 45 (interquartile range: 26) and most participants were highly educated (52.2%) (Table 1). Overall, 46.8% of our sample had one or more chronic conditions. Depression/anxiety disorder was a listed chronic condition, of which 2,699 (13.6%) participants from the total sample selected this condition (Table S1). Of those participants that have a singular chronic condition (n = 5,892), 1,248 (21.2%) have an anxiety or depression disorder (Table 1). More information on participants with chronic conditions is in the appendix (Tables S1-S2).

**Table 1:** Respondent characteristics and descriptive data on mental health for the total sample and by diagnostic *sub*-groups (*N* = 19,902)

Variable & Category	Total sample	- anxiety - depression^a^	+ anxiety - depression^a^	- anxiety + depression^a^	+ anxiety + depression^a^	*p*-value*
**Total participants [N (%)]**	19,902 (100)	14,320 (72)	1,361 (6.8)	858 (4.3)	3,363 (16.9)	
**Gender [n (%)]**						< 0.001
Male	9,294 (46.7)	7,082 (76.2)	506 (5.4)	350 (3.8)	1,356 (14.6)	
Female	10,566 (53.1)	7,223 (68.4)	853 (8.1)	504 (4.8)	1,986 (18.8)	
Other	42 (0.2)	15 (35.7)	2 (4.8)	4 (9.5)	21 (50)	
**Age group [n (%)]**						0.000
18–30	3,989 (20.0)	2,206 (55.3)	318 (8.0)	280 (7.0)	1,185 (29.7)	
31–45	6,007 (30.2)	3,944 (65.7)	506 (8.4)	279 (4.6)	1,278 (21.3)	
46–60	5,288 (26.6)	4,071 (77.0)	357 (6.8)	199 (3.8)	661 (12.5)	
61–75	4,618 (23.2)	4,099 (88.8)	180 (3.9)	100 (2.2)	239 (5.2)	
**Age (Median (IQR))**						
	45 (26)	50 (26)	41 (21)	38 (24)	35 (19)	
**Educational level [n (%)]**						
Low	2,064 (10.4)	1,509 (73.1)	137 (6.6)	90 (4.4)	328 (15.9)	0.005
Middle	7,451 (37.4)	5,239 (70.3)	524 (7.0)	355 (4.8)	1,333 (17.9)	
High	10,387 (52.2)	7,572 (72.9)	700 (6.7)	413 (4.0)	1,702 (16.4)	
**Chronic conditions [n (%)]**						
None	10,585 (53.2)	8,822 (83.3)	599 (5.7)	288 (2.7)	876 (8.3)	0.000
One or more	9,317 (46.8)	5,498 (59)	762 (8.2)	570 (6.1)	2,487 (26.7)	
**Singular chronic condition selected [n (%)]** ^b^						
Anxiety or depression	1,248 (21.2)	498 (39.9)	156 (12.5)	104 (8.3)	490 (39.3)	< 0.001
Any other	4,644 (78.8)	3,311 (71.3)	311 (6.7)	202 (4.3)	820 (17.7)	
**EQ-5D-5L – Problems on the A/D dimension [N (%)]**						0.000
None	9,848 (49.5)	9,121 (92.6)	244 (2.5)	145 (1.5)	338 (3.4)	
Slight	5,942 (29.9)	4,165 (70.1)	580 (9.8)	365 (6.1)	832 (14)	
Moderate	2,686 (13.5)	899 (33.5)	405 (15.1)	253 (9.4)	1,129 (42)	
Severe	959 (4.8)	97 (10.1)	101 (10.5)	75 (7.8)	686 (71.5)	
Extreme	467 (2.3)	38 (8.1)	31 (6.6)	20 (4.3)	378 (80.9)	
**GAD-7 ≥ 8 [n (%)]**						
Anxiety present	4,724 (23.7)	NA	1,361 (28.8)	NA	3,363 (71.2)	
Mean ± SD	4.97 ± 5.15	2.42 ± 2.46	10.26 ± 2.6	5.59 ± 1.7	13.5 ± 4.0	0.000
Min.-Max.	0–21	0–7	8–21	0–7	8–21	
None	10,869 (54.6)	10,680 (98.3)	NA	189 (1.7)	NA	
Mild	5,671 (28.5)	3,640 (64.2)	707 (12.5)	669 (11.8)	655 (11.5)	
Moderate	2,054 (10.3)	NA	549 (26.7)	NA	1,505 (73.3)	
Severe	1,308 (6.6)	NA	105 (8)	NA	1,203 (92)	
**PHQ-9 ≥ 10 [n (%)]**						
Depression present	4,221 (21.2)	NA	NA	858 (20.3)	3,363 (79.7)	
Mean ± SD	5.59 ± 6.02	2.63 ± 2.64	6.56 ± 2.2	12.5 ± 2.83	16.1 ± 4.62	0.000
Min.-Max.	0–27	0–9	0–9	10–27	10–27	
None	11,134 (55.9)	10,890 (97.8)	244 (2.2)	NA	NA	
Mild	4,547 (22.8)	3,430 (75.4)	1,117 (24.6)	NA	NA	
Moderate	2,206 (11.1)	NA	NA	698 (31.6)	1,508 (68.4)	
Moderately severe	1,250 (6.3)	NA	NA	136 (10.9)	1,114 (89.1)	
Severe	765 (3.8)	NA	NA	24 (3.1)	741 (96.9)	

Data are represented for the total population and by diagnostic sub-group, which are mutually exclusive. Percentages for the total sample are represented to 100% within each variable (by column), and percentages for the diagnostic sub-groups are represented to 100% within each category (by row). SD = standard deviation. a = Definitions of the diagnostic sub-groups (mutually exclusive groups): – anxiety – depression: GAD-7 < 8 & PHQ-9 < 10; + anxiety – depression: GAD-7 ≥ 8 & PHQ-9 < 10; – anxiety + depression: GAD-7 < 8 & PHQ-9 ≥ 10; + anxiety + depression: GAD-7 ≥ 8 & PHQ-9 ≥ 10. *One-way ANOVA test for continuous variables, chi-square for categorical variables. b = sub-sample of total population; *n* = 5,892 (100%). NA = not applicable

**Fig. 1:**
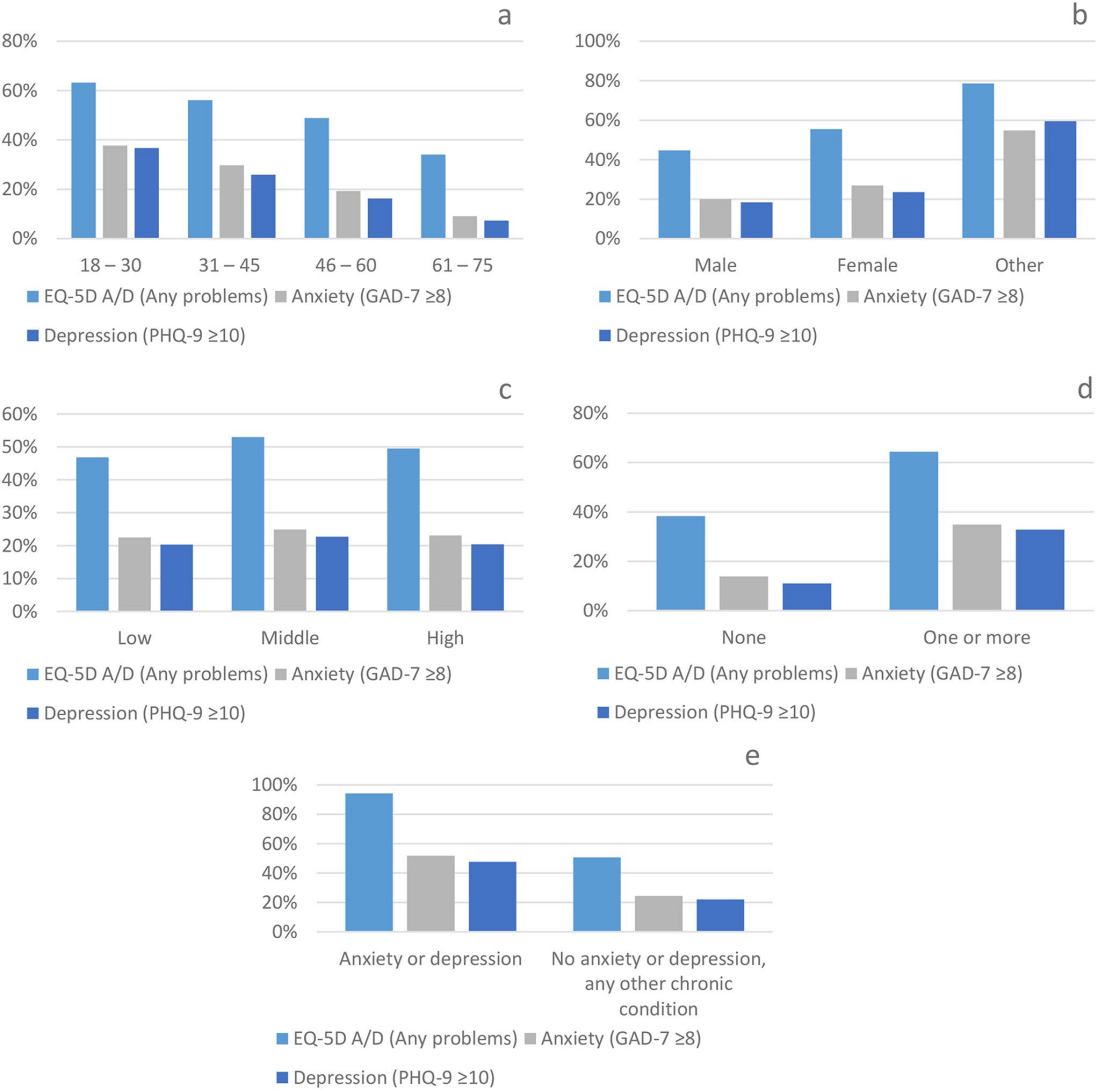
a-e Prevalence of “any problems” on the A/D dimension, and of symptoms of anxiety (GAD-7) and depression (PHQ-9) per age group (**a**), gender (**b**), education level (**c**), chronic condition (**d**) and singular chronic condition (**e**). Prevalence of any problems (slight to extreme problems) on the EQ-5D-5L Anxiety/depression dimension (vs. no problems), of anxiety symptoms (vs. no anxiety (GAD-7 < 8)) and of depression symptoms (vs. no depression (PHQ-9 < 10)) are represented per group, by percentage

On the A/D dimension, half the participants (50.5%) had slight to extreme problems (referred to as “any problems” from here on) (Table 1). Anxiety symptoms (GAD-7 ≥ 8) occurred in 4,724 (23.7%) participants, and depression symptoms (PHQ-9 ≥ 10) in 4,221 (21.2%) participants. When taking the total sample apart into mutually exclusive groups (diagnostic sub-groups), then 14,320 (72%) had no anxiety nor depression symptoms, 1,361 (6.8%) had anxiety symptoms only, 858 (4.3%) had depression symptoms only, and 3,363 (16.9%) had both anxiety and depression symptoms. By age group, any problems on the A/D dimension steadily increased from 34.1 to 63.2% with decreasing age (Fig. 1a). Similarly for symptoms of anxiety and depression, rates gradually increased from 9.1 to 37.7% and from 7.3 to 36.7%, respectively, with decreasing age. Females and other had a higher prevalence of symptoms than males (Fig. 1b), middle and highly educated had a slightly higher prevalence than low educated (Fig. 1c), those with one or more chronic conditions had around 2.5 times higher symptom prevalence on the GAD-7 and PHQ-9 than those with no chronic conditions (Fig. 1d), and those with a single chronic condition of anxiety/depression had around two times higher symptom prevalence than those with any other single chronic condition (Fig. 1e). Table 1 further illustrates the breakdown of mental health outcomes by diagnostic sub-group for each age group and problems on the A/D dimension, and provides the inclusion criteria for the diagnostic groups on the GAD-7 and PHQ-9 scales. The differences in frequencies between the diagnostic sub-groups differed significantly by sub-populations (age group, gender, educational level, chronic conditions and singular chronic condition) (*p* ≤ .005) (Table 1).

### Discriminatory performance

**Fig. 2:**
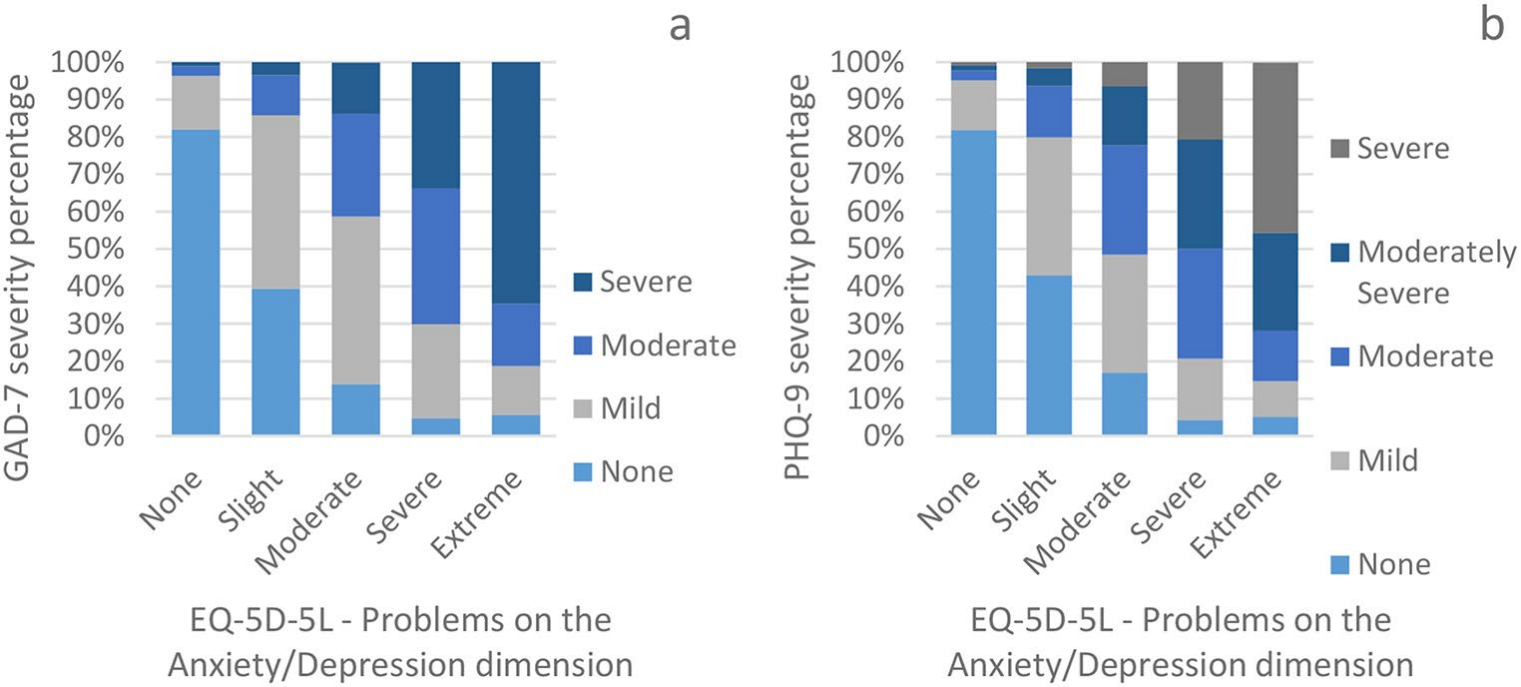
a-b Prevalence of severity of symptoms of anxiety (GAD-7 ≥ 8) (**a**) and depression (PHQ-9 ≥ 10) (**b**) compared to problems on the Anxiety/Depression dimension

As a preliminary analysis to the AUROC (Fig. 2a and b), we see that the levels of problems on the A/D dimension against the prevalence of severity on both the GAD-7 and PHQ-9 shows a steady gradient in extremities. For example, severe anxiety symptoms on the GAD-7 range from 1.1% in “no problems” on the A/D dimension to 64.7% in “extreme problems” in the total population (Fig. 2a). To support the AUROC performance analysis, the diagnostic group frequencies per sub-population are presented in Table 2. The A/D dimension performance for the total sample against the GAD-7 and PHQ-9 ranged in AUROC between 0.853 and 0.859, and did not differ significantly between diagnostic groups (Table 3). Likewise did the performance not differ significantly between the diagnostic groups within each sub-population. This was confirmed again in the non-parametric (Table S5) and higher threshold AUROC analyses (Table S6).

**Table 2:** Frequencies and proportions of diagnostic groups for the total sample and by sub-population (age group, gender, education and chronic conditions) (*N* = 19,902)

			Anxiety (GAD-7 ≥ 8)		Depression (PHQ-9 ≥ 10)		Co-morbid anxiety & depression (GAD-7 ≥ 8 & PHQ-9 ≥ 10)		
			Absent	Present	Absent	Present	Absent	Present	Total
**Total sample**		N	15,178	4,724	15,681	4,221	16,539	3,363	19,902
		%	76%	24%	79%	21%	83%	17%	100%
**Gender**									
	Male	n	7,432	1,862	7,588	1,706	7,938	1,356	9,294
		%	80%	20%	82%	18%	85%	15%	47%
	Female	n	7,727	2,839	8,076	2,490	8,580	1,986	10,566
		%	73%	27%	76%	24%	81%	19%	53%
	Other	n	19	23	17	25	21	21	42
		%	45%	55%	41%	60%	50%	50%	0%
**Age group**									
	18–30	n	2,486	1,503	2,524	1,465	2,804	1,185	3,989
		%	62%	38%	63%	37%	70%	30%	20%
	31–45	n	4,223	1,784	4,450	1,557	4,729	1,278	6,007
		%	70%	30%	74%	26%	79%	21%	30%
	46–60	n	4,270	1,018	4,428	860	4,627	661	5,288
		%	81%	19%	84%	16%	88%	13%	27%
	61–75	n	4,199	419	4,279	339	4,379	239	4,618
		%	91%	9%	93%	7%	95%	5%	23%
**Education**									
	Low	n	1,599	465	1,646	418	1,736	328	2,064
		%	78%	23%	80%	20%	84%	16%	10%
	Middle	n	5,594	1,857	5,763	1,688	6,118	1,333	7,451
		%	75%	25%	77%	23%	82%	18%	37%
	High	n	7,985	2,402	8,272	2,115	8,685	1,702	10,387
		%	77%	23%	80%	20%	84%	16%	52%
**Chronic conditions**									
	None	n	9,110	1,475	9,421	1,164	9,709	876	10,585
		%	86%	14%	89%	11%	92%	8%	53%
	One or more	n	6,068	3,249	6,260	3,057	6,830	2,487	9,317
		%	65%	35%	67%	33%	73%	27%	47%
**Singular chronic condition** ^a^		N	4,115	1,777	4,276	1,616	4,582	1,310	5,892
		%	70%	30%	73%	27%	78%	22%	100%
	Anxiety or depression	n	602	646	654	594	758	490	1,248
		%	48%	52%	52%	48%	61%	39%	21%
	Any other chronic condition	n	3,513	1,131	3,622	1,022	3,824	820	4,644
		%	76%	24%	78%	22%	82%	18%	79%

Percentages are rounded to 0 decimal points. The diagnostic groups are the non-exclusively defined groups. a = number of participants with a single chronic condition = 5,892

**Table 3:**
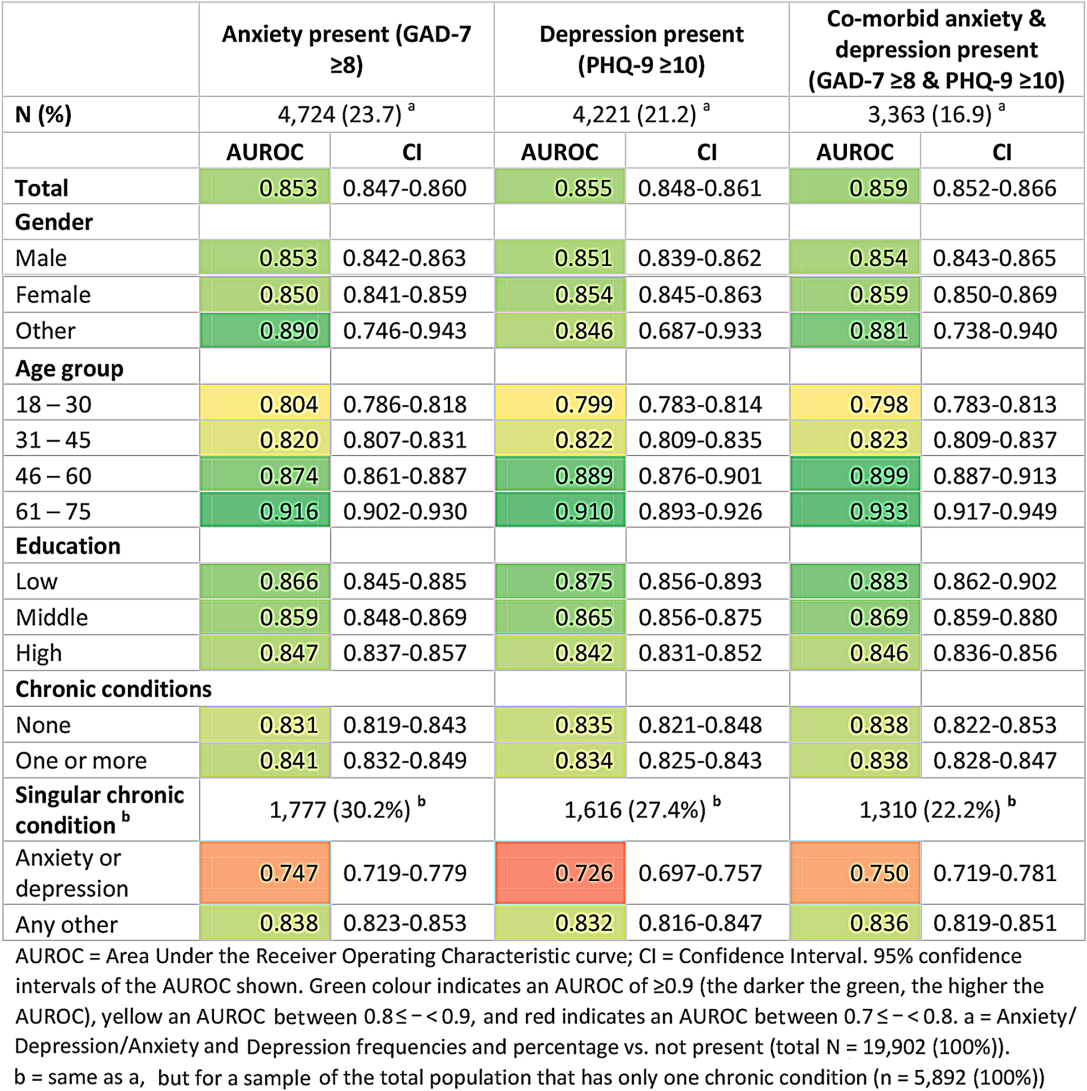
Discriminatory performance of the EQ-5D-5L A/D dimension compared to the diagnostic groups, for the total sample and by sub-populations (gender, age group, education and chronic conditions), using AUROC analysis

Across the age groups, the performance differed significantly between ages 18–30 and 31–45 versus 46–60 and 61–75 in all diagnostic groups, with AUROC ≤ 0.823 in the two younger groups and AUROC ≥ 0.874 in the two older groups (Table 3). Moreover, the AUROC was consistently ≤ 0.804 in the under-30-year-olds and ≥ 0.910 in the over-61-year-olds. The performance between groups within gender, education and chronic conditions (none vs. one or more) did not differ significantly, apart from marginally worse performance in the highly educated group compared to the low and middle educated in two of the three diagnostic groups. However, the performance did differ significantly between those with one chronic condition that is an anxiety/depression disorder and those with any other singular chronic condition. The AUROC ranged between 0.726 and 0.750 in the former group and thus presents the lowest performance among all groups, whereas the AUROC ranged between 0.832 and 0.838 for the latter group.

Given that the performance was found to differ significantly by age group and singular chronic conditions, we sought to examine whether the differences persisted by further splitting the performance analyses (Table S4). The age differences in performance largely persisted when further split by education level, most noticeably in the high education group. The stepwise differences between age groups only persisted when further split by singular chronic conditions in those with a chronic condition other than anxiety or depression. Whereas the differences in AUROC between the singular chronic conditions groups persisted more strongly in the higher age groups when comparing equals (same age groups). Furthermore, the lower versus higher performance in those having an anxiety or depression disorder versus any other persisted in the middle and high education groups only, not the low.

Using the non-parametric performance analysis yielded overall lower AUROC values, but the differences and their conclusions remained (Table S5). Similarly, the sensitivity analysis using higher thresholds for the diagnostic groups (GAD-7 ≥ 10 and PHQ-9 ≥ 15) yielded higher AUROC values, but the differences and their conclusions largely remained the same, except that the differences between ages were now more pronounced when split by education and by singular chronic condition than they were with the lower thresholds (Table S6).

### Optimal A/D dimension cut-off

Supporting frequency analyses found that the percentage of non-corresponding results on problems on the A/D dimension versus anxiety or depression symptoms was overall higher in the two younger age groups than the two older when using a cut-off on the A/D dimension of ≥ 2 (Tables S7A-C) and ≥ 3 (Tables S8A-C). The same applied in the prevalence of non-corresponding results between the singular chronic conditions groups, where this was higher for both a cut-off of ≥ 2 and ≥ 3 on the A/D dimension in the anxiety/depression group compared to the any other group (Tables S7A-C & S8A-C). The supporting data on sensitivity, specificity, PPV, NPV, accuracy and the Youden’s index show that the highest sensitivity of the A/D dimension in each of the diagnostic groups for the total sample is reached with a cut-off score of ≥ 3 for having A/D – at the cost of lower specificity, accuracy and PPV, but not NPV (Tables S9-S12). Among the two younger age groups, the better score is reached with a cut-off point on the A/D dimension of ≥ 3, as this is the highest Youden’s index of between 0.42 and 0.49 (Table S10). Contrarily, among the two older age groups, the better cut-off point is ≥ 2 (highest Youden’s index: 0.52–0.65) (Table S9). Among those with a single chronic condition that is anxiety or depression, the Youden Index of 0.32–0.37 indicates a cut-off of ≥ 4 to be the more adequate for this group (Table S11). And finally, for those with a single chronic condition other than anxiety or depression, the ideal cut-off point is between ≥ 2 or ≥ 3, as the Youden Index ranges between 0.44 and 0.47 (Tables S9-S10).

## Discussion

Our results showed that in the total sample performance analysis, the A/D dimension demonstrated good performance (AUROC > 0.85) against the GAD-7 and PHQ-9 instruments. The performance did not differ significantly between diagnostic groups, meaning the A/D dimension was not better at capturing either anxiety or depression. This statement held true when splitting the performance analysis by age group, gender, education and chronic conditions and in the sensitivity analyses. When analysing the performance by age group, significant differences were observed between those aged 18–45 and those aged 46–75, with poorer performance in the younger group. In the performance by singular chronic condition, those with only an anxiety or depression condition had significantly worse performance than those with any other singular chronic condition.

### Performance between diagnostic groups

This performance analysis study is one of two studies conducted specifically on the 5-level version of the EQ-5D’s A/D dimension in comparison to anxiety- and depression-specific screening tools [10; 11]. In one study involving participants following hospital discharge (named community setting), the performance of the 3-level version of the EQ-5D A/D dimension was evaluated against the GAD-2, PHQ-9 and both combined [10]. In comparison to this study, our AUROC results for the total sample are slightly improved, which could be a result of the improved discriminatory power of the 5-level version over the 3-level version [23]. Considering this, the AUROC values are generally comparable to those of the community setting results (AUROC: 0.78–0.86), and as in our study, did not differ significantly between instruments. In a further performance analysis study comparing the A/D dimension to the GAD-2 and PHQ-8 and both combined, slightly higher performance was detected, with improved AUROC scores in the good and excellent range [11]. This is most likely due to their study population being older on average compared to ours, which also supports our findings on the differences between the age groups (see following section). Again, the performance did not differ significantly by instrument [11]. Together with our study, these studies show that the A/D dimension is equally sensitive in picking up anxiety, depression and both anxiety and depression symptoms, exhibiting high convergent validity. Further, it is similarly sensitive across different population samples. This was not surprising, as the A/D dimension has been shown to be interpreted as taking both anxiety and depression symptoms into account in individuals self-reporting their health, compared to the Pain/Discomfort dimension where only Pain was mainly used to report on [24, 25].

### Age group performance

We observed that the A/D dimension performance was significantly improved in the older population. Moderate to good performance prevailed in the younger age groups whereas good to excellent performance was observed in the older age groups. Significant differences persisted when we additionally split the age group performance by education and chronic conditions.

These differences in performance could indicate differential item functioning (DIF) of the A/D dimension between generations. It may occur that an item of interest does not measure a construct equivalently across different groups, leading to DIF. For example, it was found that the A/D dimension exhibited age-related DIF, between older (aged 65 + years) and younger adults (aged 18–64), whereby older adults were less likely to report problems [26]. This was also found to be the case in this study, with the frequency of reporting any problems on the A/D dimension, as well as symptoms on the GAD-7 and PHQ-9, being comparatively lower in the older population compared to the younger.

### Singular chronic condition performance

We also observed significant differences in the performance of the A/D dimension between participants that have a singular chronic condition that is an anxiety or depression disorder and those that have a different singular chronic condition other than anxiety/depression. The former group had significantly worse overall performance compared to the latter group, but also compared to the other performance data. The performance values are classified as moderately accurate only, compared to good to excellent in the remaining population groups of this study. This was an unexpected finding – we would have expected the discriminatory performance to be highest in those with a (though self-reported) diagnosis of anxiety/depression disorder, exhibiting known-group validity. Studies have validated the EQ-5D instrument in diagnosed anxiety disorder or major depressive disorder (MDD) populations [27, 28], of which two found specifically the A/D dimension to be strongly correlated with other disease-specific instruments, including the GAD-7 and PHQ-9 [29, 30]. However, in the study by Supina et al., where reporting problems on the A/D dimension was compared between participants with an anxiety only diagnosis, major depressive episode only or both in a logistic regression, they concluded that there was a need for the A/D dimension to better distinguish between persons with a *single* anxiety *or* depression disorder [31]. In that study, the A/D dimension more strongly distinguished those with co-morbid anxiety and depression. However this is not reflected in the current study nor by Short et al. [10].

Given the high prevalence of 94% on any problems on the A/D dimension in the singular chronic anxiety/depression disorder group and the therewith lower prevalence of anxiety and depression symptoms on the GAD-7 (≥ 8) and PHQ-9 (≥ 10) instruments, respectively, we theorise that the lower performance has to do with medicinal treatment of symptoms in this group. We suggest that in this group the responses on the GAD-7 and PHQ-9 were lower because they were suffering less frequently from symptoms of their conditions, as they were possibly being treated with medication. Whereas responses on the A/D dimension remained high because they indeed have the condition today, but this was not considered to be related to anxiety/depression *symptoms*, but rather simply *having* the condition. Since it was a self-reported survey, no explanation of the EQ-5D instrument was provided, thus it is possible that this dimension is interpreted differently in a participant with an anxiety or depression disorder. As the evidence for this theory is limited, the interpretation in this specific group could be important to investigate.

As far as we could find, there were no studies analysing the A/D responses using a population that self-reported a chronic anxiety or depression disorder. Rather, the studies involved were either a professionally diagnosed anxiety disorder or MDD population, or a self-reporting healthy/diseased general population sample (however when diseased, not specifically having an anxiety/depression disorder).

### Strengths and limitations

This study is the largest to date to analyse the performance of the EQ-5D-5L A/D dimension, with almost 20,000 general population adults included across the majority of the lifespan. It is also the first to investigate the discriminatory performance between age groups and self-reported chronic conditions. Having said that, the EQ-5D-5L is strictly employed as a complete instrument, which was not taken into account here, as we have relied solely on the A/D dimension to capture anxiety and depression symptoms. This does not provide a complete and accurate picture of the state of anxiety and depression in the individual. All dimensions of the EQ-5D to some extent may capture symptoms of anxiety and/or depression [30; 31]. On the other hand, the A/D dimension is the primary dimension of the EQ-5D descriptive system that captures psychological symptoms [6], and as such is the most relevant in terms of representing an individual’s anxiety/depression state. This statement also reflects the fact that a person’s response heuristics to the EQ-5D questionnaire (and most other questionnaires, for that matter) is not a structured categorising of symptoms, like in Boolean logic. In the example of the A/D dimension, the Boolean logic would prescribe a ‘computational’ weighing and adding of symptoms due to the dimensions composite nature. However, we would like to stress that a person’s response does not function as the Boolean logic describes. It is more complex, multi-faceted and simply human than that.

In this analysis we could not truly determine whether differences by age and chronic conditions are indeed due to participants’ age or their conditions, or another unmeasured cohort effect. Having said that, chronic conditions are more prevalent among older adults, and we did not see significant differences in the performance between those with no chronic conditions and those with. Yet the older age groups showed improved performance. Moreover, differences persisted in all sensitivity analyses, including those split by education, and education reflects to some extent both living standards in early life and the cultural component of one’s socio-economic status [32]. Nonetheless, to validate these findings, we would advise employing an analysis strategy that can account for influencing variables that are likely to impact age and poor health, such as more nuanced living standards (e.g. GDP per capita or income) [33].

Furthermore, the performance of the A/D dimension is compared to the “gold standard” GAD-7 and PHQ-9, which are anxiety- and depression-specific and complete screening instruments. However, these two instruments are imperfect estimations, as the data are ultimately self-rated and not professional diagnoses established with a diagnostic interview. In a meta-analysis on the accuracy of the PHQ-9, it was found that the instrument may be more specific among older patients (aged 60 or over) [16]. Thus, to some extent, the differences in age may be due to the PHQ-9; however this is unlikely to explain the complete picture of differences, particularly not those in the GAD-7 diagnostic groups. This is generally the bias one encounters when investigating accuracy of self-rated questionnaires using ROC analysis, because one measure has to be regarded as the “gold standard” [34]. Having said that, these instruments have frequently proven to be accurate screening tools for the detection of generalised anxiety disorder and other anxiety disorders [35], and depression disorders cf. DSM-IV [16, 36, 37]. Our comparison is inherently imperfect as the time scales and structures are different: problems experienced “*Today”* on the A/D dimension compared to frequency of symptoms across “*two weeks”* on the GAD-7 and PHQ-9 instruments. However, in order to achieve our aim we needed to compare the EQ-5D A/D dimension with a disease-specific instrument. As the instruments’ intentions are thus different, these structural differences were unavoidable. This is likely to have affected the performance values overall, but unlikely to have affected the comparisons we make across groups.

## Conclusions

In our performance analysis of the EQ-5D-5L A/D dimension to understand the relationship between responses on the dimension and symptoms on the specific screening tools, we found that the performance was similar between diagnostic groups, thus was equally sensitive in capturing symptoms of anxiety, depression and both. Performance was worse in the younger population, possibly due to age-related differential item functioning of the A/D dimension. Performance was also worse in those having indicated anxiety/depression disorder as a chronic condition, possibly due to the lack of a description of symptoms in the A/D dimension – interpretation in this group should be further investigated. This study marks the first to analyse performance differences of the A/D dimension between groups of a general population. We present evidence that the performance of the A/D dimension may differ between generations, and thus intra-age comparative data using the EQ-5D may be flawed. We recommend further exploring these differences, given the concerning trend in mental health problems among the young population and overall.

## Electronic supplementary material

Below is the link to the electronic supplementary material.

Supplementary Material 1

## Data Availability

The original contributions presented in the study are included in the article/Supplementary Materials, further inquiries can be directed to the corresponding author/s.
