# Supplementary material for: The performance relationship between the EQ-5D-5L composite “Anxiety/Depression” dimension and anxiety and depression symptoms in a large, general population sample
Source: Qual Life Res. 2024 Sep 13;33(11):3107–19. doi: 10.1007/s11136-024-03754-5 (PMC11541259; doi:10.1007/s11136-024-03754-5)
Supplement: Supplementary file 1 — Supplementary Material 1 [file 11136_2024_3754_MOESM1_ESM.docx]

Supplementary Material to

The performance relationship between the EQ-5D-5L composite “anxiety/depression” dimension and anxiety and depression symptoms in a large, general population sample

**Emily Stella Scott^1^*, Erica I. Lubetkin^2^, Mathieu F. Janssen^3^, John N. Yfantopolous^4^, Gouke J. Bonsel^5^, Juanita A. Haagsma^1^**

^1^ Department of Public Health, Erasmus MC, Rotterdam, The Netherlands

^2^ Department of Community Health and Social Medicine, CUNY School of Medicine, New York City, New York, USA

^3^ Section Medical Psychology and Psychotherapy, Department of Psychiatry, Erasmus MC, Rotterdam, The Netherlands

^4^ Health Department of Economics, National and Kapodistrian University of Athens, Athens, Greece

^5^ EuroQol Research Foundation, Rotterdam, The Netherlands

* Corresponding author: Emily Stella Scott, ORCID: 0000-0001-7548-1775
[e.scott@erasmusmc.nl](mailto:e.scott@erasmusmc.nl)

Supplementary Material

Table S1. Frequencies of chronic conditions (N=19,902).

| Chronic condition | N selected | % of total sample |
| --- | --- | --- |
| Asthma; chronic bronchitis | 2,124 | 10.7% |
| Heart disease | 1,058 | 5.3% |
| Consequences of a stroke | 369 | 1.9% |
| Diabetes | 1,753 | 8.8% |
| Chronic rheumatoid arthritis | 656 | 3.3% |
| Severe back complaints/arthrosis of the back | 1,410 | 7.1% |
| Painful/swollen joints of knee or hip due to arthrosis | 1,384 | 7.0% |
| Cancer | 435 | 2.2% |
| Memory problems due to a neurological disease/dementia | 476 | 2.4% |
| Memory problems due to ageing | 434 | 2.2% |
| Depression or anxiety disorder | 2,699 | 13.6% |
| Other | 2,215 | 11.1% |

Table S2. Chronic condition anxiety or depression disorder vs. symptoms of anxiety or depression (as measured by GAD-7 and PHQ-9), by diagnostic sub-groups.

| Depression or anxiety disorder as a chronic health condition | | Diagnostic sub-group | | | | Total |
| --- | --- | --- | --- | --- | --- | --- |
|  |  | - anxiety  - depression ^a^ | + anxiety  - depression ^a^ | - anxiety  + depression ^a^ | + anxiety  + depression ^a^ |  |
| No | n | 13,407 | 1,064 | 606 | 2,126 | 17,203 |
|  | % | 77.9% | 6.2% | 3.5% | 12.4% | 100.0% |
| Yes | n | 913 | 297 | 252 | 1,237 | 2,699 |
|  | % | 33.8% | 11.0% | 9.3% | 45.8% | 100.0% |
| Total | N | 14,320 | 1,361 | 858 | 3,363 | 19,902 |
|  | % | 72.0% | 6.8% | 4.3% | 16.9% | 100.0% |

a = Definitions of the diagnostic sub-groups (mutually exclusive groups): – anxiety – depression: GAD-7 <8 & PHQ-9 <10; + anxiety – depression: GAD-7 ≥8 & PHQ-9 <10; – anxiety + depression: GAD-7 <8 & PHQ-9 ≥10; + anxiety + depression: GAD-7 ≥8 & PHQ-9 ≥10. Percentages are rounded to 1 decimal point.

Table S3. Supporting frequencies and proportions for Table S4 per diagnostic group for the total sample, by age group (binary), education & age groups (binary) and for those with a singular chronic condition, by singular chronic condition & age groups (binary), and by education & singular chronic condition.

|  |  |  | Anxiety (GAD-7 ≥8) | | Depression (PHQ-9 ≥10) | | Co-morbid anxiety & depression (GAD-7 ≥8 & PHQ-9 ≥10) | | Total |
| --- | --- | --- | --- | --- | --- | --- | --- | --- | --- |
|  |  |  | Absent | Present | Absent | Present | Absent | Present |  |
| Age group (binary) ^a^ | | | | | | | | | |
|  | 18-45 | n | 6,709 | 3,287 | 6,974 | 3,022 | 7,533 | 2,463 | 9,996 |
|  |  | % | 44% | 70% | 45% | 72% | 46% | 73% | 50% |
|  | 46-75 | n | 8,469 | 1,437 | 8,707 | 1,199 | 9,006 | 900 | 9,906 |
|  |  | % | 56% | 30% | 56% | 28% | 55% | 27% | 50% |
| Education & Age group ^a^ | | | | | | | | | |
| Low | 18-30 | n | 191 | 123 | 186 | 128 | 211 | 103 | 314 |
|  |  | % | 12% | 27% | 11% | 31% | 12% | 31% | 15% |
|  | 31-45 | n | 263 | 149 | 272 | 140 | 295 | 117 | 412 |
|  |  | % | 16% | 32% | 17% | 34% | 17% | 36% | 20% |
|  | 46-60 | n | 494 | 117 | 516 | 95 | 545 | 66 | 611 |
|  |  | % | 31% | 25% | 31% | 23% | 31% | 20% | 30% |
|  | 61-75 | n | 651 | 76 | 672 | 55 | 685 | 42 | 727 |
|  |  | % | 41% | 16% | 41% | 13% | 40% | 13% | 35% |
| Middle | 18-30 | n | 983 | 652 | 990 | 645 | 1,114 | 521 | 1,635 |
|  |  | % | 18% | 35% | 17% | 38% | 18% | 39% | 22% |
|  | 31-45 | n | 1,321 | 591 | 1400 | 512 | 1,503 | 409 | 1,912 |
|  |  | % | 24% | 32% | 24% | 30% | 25% | 31% | 26% |
|  | 46-60 | n | 1,687 | 444 | 1739 | 392 | 1,827 | 304 | 2,131 |
|  |  | % | 30% | 24% | 30% | 23% | 30% | 23% | 29% |
|  | 61-75 | n | 1,603 | 170 | 1634 | 139 | 1,674 | 99 | 1,773 |
|  |  | % | 29% | 9% | 28% | 8% | 27% | 7% | 24% |
| High | 18-30 | n | 1,312 | 728 | 1348 | 692 | 1,479 | 561 | 2,040 |
|  |  | % | 16% | 30% | 16% | 33% | 17% | 33% | 20% |
|  | 31-45 | n | 2,639 | 1,044 | 2778 | 905 | 2,931 | 752 | 3,683 |
|  |  | % | 33% | 44% | 34% | 43% | 34% | 44% | 36% |
|  | 46-60 | n | 2,089 | 457 | 2173 | 373 | 2,255 | 291 | 2,546 |
|  |  | % | 26% | 19% | 26% | 18% | 26% | 17% | 25% |
|  | 61-75 | n | 1,945 | 173 | 1973 | 145 | 2,020 | 98 | 2,118 |
|  |  | % | 24% | 7% | 24% | 7% | 23% | 6% | 20% |
| Education & Age group (binary) ^a^ | | | | | | | | | |
| Low | 18-45 | n | 454 | 272 | 458 | 268 | 506 | 220 | 726 |
|  |  | % | 28% | 59% | 28% | 64% | 29% | 67% | 35% |
|  | 46-75 | n | 1,145 | 193 | 1,188 | 150 | 1,230 | 108 | 1,338 |
|  |  | % | 72% | 42% | 72% | 36% | 71% | 33% | 65% |
| Middle | 18-45 | n | 2,304 | 1,243 | 2,390 | 1,157 | 2,617 | 930 | 3,547 |
|  |  | % | 41% | 67% | 42% | 69% | 43% | 70% | 48% |
|  | 46-75 | n | 3,290 | 614 | 3,373 | 531 | 3,501 | 403 | 3,904 |
|  |  | % | 59% | 33% | 59% | 32% | 57% | 30% | 52% |
| High | 18-45 | n | 3,951 | 1,772 | 4,126 | 1,597 | 4,410 | 1,313 | 5,723 |
|  |  | % | 50% | 74% | 50% | 76% | 51% | 77% | 55% |
|  | 46-75 | n | 4,034 | 630 | 4,146 | 518 | 4,275 | 389 | 4,664 |
|  |  | % | 51% | 26% | 50% | 25% | 49% | 23% | 45% |
| Singular chronic condition & Age group ^b^ | | | | | | | | | |
| Anxiety or depression | 18-30 | n | 163 | 255 | 176 | 242 | 212 | 206 | 418 |
|  |  | % | 27% | 40% | 27% | 41% | 28% | 42% | 34% |
|  | 31-45 | n | 225 | 238 | 247 | 216 | 291 | 172 | 463 |
|  |  | % | 37% | 37% | 38% | 36% | 38% | 35% | 37% |
|  | 46-60 | n | 155 | 124 | 168 | 111 | 184 | 95 | 279 |
|  |  | % | 26% | 19% | 26% | 19% | 24% | 19% | 22% |
|  | 61-75 | n | 59 | 29 | 63 | 25 | 71 | 17 | 88 |
|  |  | % | 10% | 5% | 10% | 4% | 9% | 4% | 7% |
| Any other chronic condition | 18-30 | n | 410 | 411 | 411 | 410 | 473 | 348 | 821 |
|  |  | % | 12% | 36% | 11% | 40% | 12% | 42% | 18% |
|  | 31-45 | n | 742 | 412 | 773 | 381 | 849 | 305 | 1154 |
|  |  | % | 21% | 36% | 21% | 37% | 22% | 37% | 25% |
|  | 46-60 | n | 1022 | 218 | 1066 | 174 | 1116 | 124 | 1240 |
|  |  | % | 29% | 19% | 29% | 17% | 29% | 15% | 27% |
|  | 61-75 | n | 1339 | 90 | 1372 | 57 | 1386 | 43 | 1429 |
|  |  | % | 38% | 8% | 38% | 6% | 36% | 5% | 31% |
| Singular chronic condition & Age group (binary) ^b^ | | | | | | | | | |
| Anxiety or depression | 18-45 | n | 388 | 493 | 423 | 458 | 503 | 378 | 881 |
|  |  | % | 65% | 76% | 65% | 77% | 66% | 77% | 71% |
|  | 46-75 | n | 214 | 153 | 231 | 136 | 255 | 112 | 367 |
|  |  | % | 36% | 24% | 35% | 23% | 34% | 23% | 29% |
| Any other chronic condition | 18-45 | n | 1,152 | 823 | 1184 | 791 | 1322 | 653 | 1975 |
|  |  | % | 33% | 73% | 33% | 77% | 35% | 80% | 43% |
|  | 46-75 | n | 2,361 | 308 | 2438 | 231 | 2502 | 167 | 2669 |
|  |  | % | 67% | 27% | 67% | 23% | 65% | 20% | 58% |
| Education & Singular chronic condition ^b^ | | | | | | | | | |
| Low | Anxiety or depression | n | 60 | 65 | 66 | 59 | 75 | 50 | 125 |
|  |  | % | 12% | 36% | 13% | 36% | 13% | 38% | 18% |
|  | Any other | n | 446 | 118 | 458 | 106 | 483 | 81 | 564 |
|  |  | % | 88% | 65% | 87% | 64% | 87% | 62% | 82% |
| Middle | Anxiety or depression | n | 226 | 276 | 250 | 252 | 295 | 207 | 502 |
|  |  | % | 14% | 40% | 15% | 39% | 17% | 41% | 22% |
|  | Any other | n | 1350 | 411 | 1373 | 388 | 1463 | 298 | 1761 |
|  |  | % | 86% | 60% | 85% | 61% | 83% | 59% | 78% |
| High | Anxiety or depression | n | 316 | 305 | 338 | 283 | 388 | 233 | 621 |
|  |  | % | 16% | 34% | 16% | 35% | 17% | 35% | 21% |
|  | Any other | n | 1717 | 602 | 1791 | 528 | 1878 | 441 | 2319 |
|  |  | % | 85% | 66% | 84% | 65% | 83% | 65% | 79% |

a = Anxiety/Depression/Anxiety and Depression frequencies and percentage vs. not present (total N = 19,902 (100%)). b = same as a, but for a sample of the total population that has only one chronic condition (n = 5,892 (100%)).

Table S4. Performance of the EQ-5D-5L A/D dimension compared to the diagnostic groups (GAD-7 and/or PHQ-9) in measuring anxiety, depression, both and none; by age groups, education and age, singular chronic condition and age and singular chronic condition and education, using the **parametric** Area under the Receiver Operating characteristic Curve (AUROC).

|  | | **Anxiety present (GAD-7 ≥8)** | | | | **Depression present (PHQ-9 ≥10)** | | | **Co-morbid anxiety & depression present (GAD-7 ≥8 & PHQ-9 ≥10)** | |
| --- | --- | --- | --- | --- | --- | --- | --- | --- | --- | --- |
| **N (%)** | | **4,724 (23.7) ^a^** | | | | **4,221 (21.2) ^a^** | | | **3,363 (16.9) ^a^** | |
|  | | AUROC | | CI | | AUROC | CI | | AUROC | CI |
| **Age group (binary)** | | | | | | | | | | |
| 18 – 45 | | 0.815 | | 0.805-0.824 | | 0.814 | 0.805-0.824 | | 0.814 | 0.804-0.824 |
| 46 – 75 | | 0.895 | | 0.885-0.904 | | 0.902 | 0.893-0.911 | | 0.916 | 0.906-0.925 |
| **Education & Age group** | | | | | | | | | | |
| Low | 18 – 30 | 0.813 | | 0.760-0.859 | | 0.814 | 0.764-0.863 | | 0.801 | 0.747-0.854 |
|  | 31 – 45 | 0.808 | | 0.759-0.852 | | 0.825 | 0.780-0.868 | | 0.834 | 0.787-0.877 |
|  | 46 – 60 | 0.864 | | 0.823-0.900 | | 0.897 | 0.862-0.929 | | 0.918 | 0.882-0.953 |
|  | 61 – 75 | 0.925 | | 0.899-0.948 | | 0.918 | 0.879-0.952 | | 0.949 | 0.915-0.973 |
| Middle | 18 – 30 | 0.815 | | 0.795-0.838 | | 0.816 | 0.794-0.838 | | 0.810 | 0.787-0.834 |
|  | 31 – 45 | 0.827 | | 0.805-0.848 | | 0.832 | 0.810-0.855 | | 0.835 | 0.811-0.858 |
|  | 46 – 60 | 0.876 | | 0.856-0.896 | | 0.888 | 0.870-0.907 | | 0.904 | 0.886-0.923 |
|  | 61 – 75 | 0.897 | | 0.872-0.923 | | 0.912 | 0.887-0.938 | | 0.930 | 0.902-0.955 |
| High | 18 – 30 | 0.792 | | 0.772-0.813 | | 0.780 | 0.757-0.802 | | 0.787 | 0.763-0.809 |
|  | 31 – 45 | 0.817 | | 0.799-0.833 | | 0.816 | 0.798-0.833 | | 0.816 | 0.797-0.834 |
|  | 46 – 60 | 0.876 | | 0.857-0.894 | | 0.886 | 0.866-0.906 | | 0.890 | 0.869-0.911 |
|  | 61 – 75 | 0.928 | | 0.907-0.946 | | 0.907 | 0.881-0.931 | | 0.933 | 0.909-0.956 |
| **Education & Age group (binary)** | | | | | | | | | | |
| Low | 18 – 45 | 0.808 | | 0.775-0.841 | | 0.820 | 0.786-0.853 | | 0.819 | 0.785-0.852 |
|  | 46 – 75 | 0.897 | | 0.869-0.919 | | 0.910 | 0.882-0.932 | | 0.933 | 0.910-0.955 |
| Middle | 18 – 45 | 0.823 | | 0.807-0.838 | | 0.825 | 0.811-0.840 | | 0.824 | 0.808-0.840 |
|  | 46 – 75 | 0.889 | | 0.874-0.904 | | 0.903 | 0.889-0.916 | | 0.918 | 0.904-0.934 |
| High | 18 – 45 | 0.810 | | 0.797-0.823 | | 0.804 | 0.790-0.817 | | 0.807 | 0.793-0.821 |
|  | 46 – 75 | 0.898 | | 0.883-0.912 | | 0.898 | 0.883-0.912 | | 0.909 | 0.894-0.924 |
| **Singular chronic condition & Age group ^b^** | | **1,777 (30.2%) ^b^** | | | | **1,616 (27.4%) ^b^** | | | **1,310 (22.2%) ^b^** | |
| Anxiety or depression | 18 – 30 | 0.732 | | 0.681-0.787 | | 0.680 | 0.624-0.740 | | 0.715 | 0.660-0.772 |
|  | 31 – 45 | 0.737 | | 0.689-0.789 | | 0.741 | 0.693-0.794 | | 0.760 | 0.710-0.815 |
|  | 46 – 60 | 0.753 | | 0.690-0.820 | | 0.765 | 0.699-0.822 | | 0.761 | 0.694-0.833 |
|  | 61 – 75 | 0.804 | | 0.678-0.893 | | 0.726 | 0.573-0.852 | | 0.747 | 0.559-0.900 |
| Any other | 18 – 30 | 0.782 | | 0.747-0.814 | | 0.763 | 0.728-0.797 | | 0.756 | 0.723-0.792 |
|  | 31 – 45 | 0.770 | | 0.739-0.803 | | 0.772 | 0.742-0.803 | | 0.769 | 0.734-0.802 |
|  | 46 – 60 | 0.825 | | 0.793-0.856 | | 0.833 | 0.798-0.868 | | 0.861 | 0.827-0.896 |
|  | 61 – 75 | 0.913 | | 0.876-0.942 | | 0.908 | 0.860-0.947 | | 0.937 | 0.893-0.963 |
| **Singular chronic condition & Age group (binary) ^b^** | | | | | | | | | | |
| Anxiety or depression | 18 – 45 | 0.735 | 0.700-0.773 | | 0.711 | | 0.675-0.751 | 0.737 | | 0.701-0.777 |
|  | 46 – 75 | 0.768 | 0.712-0.825 | | 0.755 | | 0.695-0.813 | 0.775 | | 0.715-0.838 |
| Any other | 18 – 45 | 0.781 | 0.758-0.802 | | 0.770 | | 0.748-0.792 | 0.766 | | 0.743-0.789 |
|  | 46 – 75 | 0.870 | 0.847-0.892 | | 0.869 | | 0.843-0.896 | 0.899 | | 0.874-0.923 |
| **Education & Singular chronic condition ^b^** | | | | | | | | | | |
| Low | Anxiety or depression | 0.782 | 0.685-0.852 | | 0.786 | | 0.690-0.859 | 0.807 | | 0.719-0.893 |
|  | Any other | 0.831 | 0.781-0.879 | | 0.841 | | 0.784-0.886 | 0.854 | | 0.798-0.902 |
| Middle | Anxiety or depression | 0.767 | 0.721-0.816 | | 0.725 | | 0.678-0.780 | 0.757 | | 0.708-0.799 |
|  | Any other | 0.848 | 0.824-0.8670 | | 0.842 | | 0.819-0.864 | 0.848 | | 0.825-0.871 |
| High | Anxiety or depression | 0.723 | 0.680-0.769 | | 0.715 | | 0.670-0.762 | 0.734 | | 0.689-0.782 |
|  | Any other | 0.833 | 0.811-0.851 | | 0.821 | | 0.796-0.844 | 0.823 | | 0.800-0.847 |

AUROC = Area Under the Receiver Operating Characteristic curve; CI = Confidence Interval. 95% confidence intervals of the AUROC shown. Green colour indicates an AUROC of ≥0.9 (the darker the green, the higher the AUROC), yellow an AUROC of 0.8 ≤ - < 0.9, and red indicates an AUROC of 0.7 ≤ - < 0.8. a = Anxiety/Depression/Anxiety and Depression frequencies and percentage vs. not present (total N = 19,902 (100%)). b = same as a, but for a sample of the total population that has only one chronic condition (n = 5,892 (100%)).

Table S5. Performance of the EQ-5D-5L A/D dimension compared to the diagnostic groups (GAD-7 and/or PHQ-9) in measuring anxiety, depression, both and none; by sub-populations (age group, gender, education, chronic conditions and singular chronic conditions) as well as further splitting of the sub-populations, using the **non-parametric** Area under the Receiver Operating characteristic Curve (AUROC).

|  |  | **Anxiety present (GAD-7 ≥8)** | | **Depression present (PHQ-9 ≥10)** | | **Co-morbid anxiety & depression present (GAD-7 ≥8 & PHQ-9 ≥10)** | |
| --- | --- | --- | --- | --- | --- | --- | --- |
| **N (%)** | | **4,724 (23.7) ^a^** | | **4,221 (21.2) ^a^** | | **3,363 (16.9) ^a^** | |
|  |  | AUROC | CI | AUROC | CI | AUROC | CI |
| **Total** | | 0.824 | 0.817-0.830 | 0.827 | 0.820-0.834 | 0.835 | 0.828-0.843 |
| **Gender** | |  |  |  |  |  |  |
| Male | | 0.818 | 0.807-0.829 | 0.819 | 0.808-0.830 | 0.825 | 0.813-0.837 |
| Female | | 0.824 | 0.815-0.832 | 0.829 | 0.820-0.839 | 0.840 | 0.830-0.849 |
| Other | | 0.874 | 0.773-0.975 | 0.841 | 0.707-0.975 | 0.867 | 0.759-0.976 |
| **Age bracket** | |  |  |  |  |  |  |
| 18 – 30 | | 0.781 | 0.767-0.795 | 0.776 | 0.761-0.790 | 0.780 | 0.764-0.795 |
| 31 – 45 | | 0.793 | 0.780-0.805 | 0.798 | 0.785-0.811 | 0.802 | 0.788-0.816 |
| 46 – 60 | | 0.846 | 0.833-0.859 | 0.865 | 0.852-0.879 | 0.879 | 0.866-0.893 |
| 61 – 75 | | 0.882 | 0.866-0.899 | 0.879 | 0.859-0.898 | 0.909 | 0.890-0.927 |
| **Education** | |  |  |  |  |  |  |
| Low | | 0.836 | 0.816-0.857 | 0.847 | 0.826-0.868 | 0.861 | 0.839-0.883 |
| Middle | | 0.832 | 0.822-0.843 | 0.840 | 0.829-0.851 | 0.848 | 0.837-0.859 |
| High | | 0.814 | 0.804-0.824 | 0.812 | 0.802-0.823 | 0.820 | 0.809-0.831 |
| **Chronic conditions** | |  |  |  |  |  |  |
| None | | 0.783 | 0.770-0.795 | 0.791 | 0.777-0.805 | 0.798 | 0.782-0.814 |
| One or more | | 0.819 | 0.811-0.828 | 0.813 | 0.804-0.822 | 0.820 | 0.811-0.830 |
| **Singular chronic condition ^b^** | | **1,777 (30.2%) ^b^** | | **1,616 (27.4%) ^b^** | | **1,310 (22.2%) ^b^** | |
| Anxiety or depression | | 0.737 | 0.711-0.763 | 0.719 | 0.692-0.746 | 0.737 | 0.710-0.764 |
| Any other | | 0.803 | 0.788-0.817 | 0.799 | 0.784-0.814 | 0.807 | 0.790-0.823 |
| **Age group (binary)** | |  |  |  |  |  |  |
| 18 – 45 |  | 0.815 | 0.805-0.824 | 0.814 | 0.805-0.824 | 0.814 | 0.804-0.824 |
| 46 – 75 |  | 0.895 | 0.885-0.904 | 0.902 | 0.893-0.911 | 0.916 | 0.906-0.925 |
| **Education & Age group** | | |  |  |  |  |  |
| Low | 18 – 30 | 0.813 | 0.760-0.859 | 0.814 | 0.764-0.863 | 0.801 | 0.747-0.854 |
|  | 31 – 45 | 0.808 | 0.759-0.852 | 0.825 | 0.780-0.868 | 0.834 | 0.787-0.877 |
|  | 46 – 60 | 0.864 | 0.823-0.900 | 0.897 | 0.862-0.929 | 0.918 | 0.882-0.953 |
|  | 61 – 75 | 0.925 | 0.899-0.948 | 0.918 | 0.879-0.952 | 0.949 | 0.915-0.973 |
| Middle | 18 – 30 | 0.815 | 0.795-0.838 | 0.816 | 0.794-0.838 | 0.810 | 0.787-0.834 |
|  | 31 – 45 | 0.827 | 0.805-0.848 | 0.832 | 0.810-0.855 | 0.835 | 0.811-0.858 |
|  | 46 – 60 | 0.876 | 0.856-0.896 | 0.888 | 0.870-0.907 | 0.904 | 0.886-0.923 |
|  | 61 – 75 | 0.897 | 0.872-0.923 | 0.912 | 0.887-0.938 | 0.930 | 0.902-0.955 |
| High | 18 – 30 | 0.792 | 0.772-0.813 | 0.780 | 0.757-0.802 | 0.787 | 0.763-0.809 |
|  | 31 – 45 | 0.817 | 0.799-0.833 | 0.816 | 0.798-0.833 | 0.816 | 0.797-0.834 |
|  | 46 – 60 | 0.876 | 0.857-0.894 | 0.886 | 0.866-0.906 | 0.890 | 0.869-0.911 |
|  | 61 – 75 | 0.928 | 0.907-0.946 | 0.907 | 0.881-0.931 | 0.933 | 0.909-0.956 |
| **Education & Age group (binary)** | | |  |  |  |  |  |
| Low | 18 – 45 | 0.808 | 0.775-0.841 | 0.820 | 0.786-0.853 | 0.819 | 0.785-0.852 |
|  | 46 – 75 | 0.897 | 0.869-0.919 | 0.910 | 0.882-0.932 | 0.933 | 0.910-0.955 |
| Middle | 18 – 45 | 0.823 | 0.807-0.838 | 0.825 | 0.811-0.840 | 0.824 | 0.808-0.840 |
|  | 46 – 75 | 0.889 | 0.874-0.904 | 0.903 | 0.889-0.916 | 0.918 | 0.904-0.934 |
| High | 18 – 45 | 0.810 | 0.797-0.823 | 0.804 | 0.790-0.817 | 0.807 | 0.793-0.821 |
|  | 46 – 75 | 0.898 | 0.883-0.912 | 0.898 | 0.883-0.912 | 0.909 | 0.894-0.924 |
| **Singular chronic condition & Age group ^b^** | | | | | | | |
| Anxiety or depression | 18 – 30 | 0.719 | 0.673-0.766 | 0.673 | 0.624-0.722 | 0.694 | 0.646-0.742 |
|  | 31 – 45 | 0.732 | 0.689-0.774 | 0.729 | 0.685-0.772 | 0.751 | 0.707-0.796 |
|  | 46 – 60 | 0.744 | 0.688-0.801 | 0.759 | 0.703-0.814 | 0.766 | 0.708-0.824 |
|  | 61 – 75 | 0.774 | 0.681-0.867 | 0.713 | 0.588-0.839 | 0.755 | 0.618-0.891 |
| Any other | 18 – 30 | 0.757 | 0.726-0.788 | 0.739 | 0.707-0.771 | 0.738 | 0.705-0.771 |
|  | 31 – 45 | 0.751 | 0.722-0.780 | 0.749 | 0.720-0.778 | 0.751 | 0.719-0.784 |
|  | 46 – 60 | 0.788 | 0.757-0.819 | 0.804 | 0.769-0.839 | 0.831 | 0.796-0.866 |
|  | 61 – 75 | 0.871 | 0.834-0.907 | 0.869 | 0.822-0.915 | 0.904 | 0.861-0.948 |
| **Singular chronic condition & Age group (binary) ^b^** | | | | | | | |
| Anxiety or depression | 18 – 45 | 0.726 | 0.695-0.757 | 0.702 | 0.670-0.735 | 0.722 | 0.690-0.755 |
|  | 46 – 75 | 0.752 | 0.703-0.8 | 0.751 | 0.701-0.802 | 0.766 | 0.713-0.818 |
| Any other | 18 – 45 | 0.756 | 0.735-0.777 | 0.746 | 0.725-0.767 | 0.747 | 0.725-0.770 |
|  | 46 – 75 | 0.829 | 0.806-0.852 | 0.836 | 0.809-0.863 | 0.867 | 0.840-0.894 |
| **Education & Singular chronic condition ^b^** | | | | | | | |
| Low | Anxiety or depression | 0.758 | 0.679-0.838 | 0.764 | 0.684-0.845 | 0.800 | 0.723-0.877 |
|  | Any other | 0.799 | 0.752-0.845 | 0.805 | 0.757-0.853 | 0.826 | 0.773-0.879 |
| Middle | Anxiety or depression | 0.742 | 0.702-0.782 | 0.710 | 0.668-0.753 | 0.723 | 0.679-0.766 |
|  | Any other | 0.811 | 0.788-0.835 | 0.807 | 0.783-0.830 | 0.817 | 0.791-0.843 |
| High | Anxiety or depression | 0.726 | 0.688-0.764 | 0.714 | 0.675-0.753 | 0.734 | 0.694-0.774 |
|  | Any other | 0.797 | 0.776-0.817 | 0.791 | 0.769-0.813 | 0.794 | 0.771-0.818 |

AUROC = Area Under the Receiver Operating Characteristic curve; CI = Confidence Interval. 95% confidence intervals of the AUROC shown. Green colour indicates an AUROC of ≥0.9 (the darker the green, the higher the AUROC), yellow an AUROC of 0.8 ≤ - < 0.9, and red indicates an AUROC of 0.7 ≤ - < 0.8. a = Anxiety/Depression/Anxiety and Depression frequencies and percentage vs. not present (total N = 19,902 (100%)). b = same as a, but for a sample of the total population that has only one chronic condition (n = 5,892 (100%)).

Table S6. Performance of the EQ-5D-5L A/D dimension compared to the **higher threshold diagnostic groups (GAD-7 and/or PHQ-9)** in measuring anxiety, depression, both and none; by sub-populations (age group, gender, education, chronic conditions and singular chronic conditions) as well as further splitting of the sub-populations, using the **parametric** Area under the Receiver Operating characteristic Curve (AUROC).

|  |  | **Anxiety present (GAD-7 ≥10)** | | **Depression present (PHQ-9 ≥15)** | | **Co-morbid anxiety & depression present (GAD-7 ≥10 & PHQ-9 ≥15)** | |
| --- | --- | --- | --- | --- | --- | --- | --- |
| **N (%)** | | 3,362 (16.9) ^a^ | | 2,015 (10.1) ^a^ | | 1,682 (8.5) ^a^ | |
|  |  | **AUROC** | **CI** | **AUROC** | **CI** | **AUROC** | **CI** |
| **Total** | | 0.853 | 0.845-0.860 | 0.848 | 0.838-0.857 | 0.851 | 0.841-0.862 |
| **Age bracket** | |  |  |  |  |  |  |
| 18 – 30 | | 0.794 | 0.778-0.810 | 0.777 | 0.756-0.798 | 0.780 | 0.756-0.802 |
| 31 – 45 | | 0.819 | 0.805-0.833 | 0.809 | 0.791-0.827 | 0.813 | 0.794-0.831 |
| 46 – 60 | | 0.881 | 0.866-0.895 | 0.898 | 0.881-0.914 | 0.905 | 0.887-0.922 |
| 61 – 75 | | 0.920 | 0.902-0.936 | 0.937 | 0.916-0.957 | 0.944 | 0.921-0.964 |
| **Gender** | |  |  |  |  |  |  |
| Male | | 0.850 | 0.837-0.861 | 0.836 | 0.820-0.852 | 0.836 | 0.818-0.854 |
| Female | | 0.852 | 0.842-0.861 | 0.853 | 0.841-0.865 | 0.859 | 0.847-0.872 |
| Other | | 0.827 | 0.664-0.918 | 0.808 | 0.647-0.920 | 0.858 | 0.660-0.951 |
| **Education** | |  |  |  |  |  |  |
| Low | | 0.859 | 0.836-0.880 | 0.858 | 0.829-0.886 | 0.865 | 0.831-0.896 |
| Middle | | 0.864 | 0.854-0.876 | 0.874 | 0.861-0.887 | 0.880 | 0.866-0.894 |
| High | | 0.843 | 0.831-0.853 | 0.825 | 0.810-0.840 | 0.827 | 0.810-0.842 |
| **Chronic conditions** | |  |  |  |  |  |  |
| None | | 0.836 | 0.821-0.850 | 0.811 | 0.785-0.833 | 0.817 | 0.790-0.841 |
| One or more | | 0.835 | 0.826-0.845 | 0.828 | 0.815-0.840 | 0.831 | 0.818-0.844 |
| **Singular chronic condition ^b^** | | **1,777 (30.2%) ^b^** | | **1,616 (27.4%) ^b^** | | **1,310 (22.2%) ^b^** | |
| Anxiety or depression | | 0.753 | 0.722-0.786 | 0.760 | 0.726-0.798 | 0.765 | 0.730-0.801 |
| Any other chronic condition | | 0.824 | 0.806-0.841 | 0.808 | 0.785-0.831 | 0.811 | 0.785-0.838 |
| **Age group (binary)** | |  |  |  |  |  |  |
| 18 – 45 |  | 0.810 | 0.800-0.820 | 0.797 | 0.784-0.810 | 0.800 | 0.786-0.814 |
| 46 – 75 |  | 0.900 | 0.890-0.910 | 0.916 | 0.904-0.928 | 0.923 | 0.910-0.936 |
| **Education & Age group** | | |  |  |  |  |  |
| Low | 18 – 30 | 0.783 | 0.719-0.838 | 0.783 | 0.714-0.850 | 0.754 | 0.667-0.832 |
|  | 31 – 45 | 0.810 | 0.759-0.860 | 0.782 | 0.719-0.840 | 0.809 | 0.743-0.869 |
|  | 46 – 60 | 0.894 | 0.876-0.913 | 0.911 | 0.890-0.933 | 0.920 | 0.899-0.940 |
|  | 61 – 75 | 0.951 | 0.837-0.958 | 0.921 | 0.858-0.971 | 0.945 | 0.881-0.990 |
| Middle | 18 – 30 | 0.817 | 0.793-0.840 | 0.792 | 0.761-0.823 | 0.800 | 0.768-0.831 |
|  | 31 – 45 | 0.824 | 0.799-0.847 | 0.857 | 0.830-0.884 | 0.860 | 0.831-0.888 |
|  | 46 – 60 | 0.894 | 0.876-0.913 | 0.911 | 0.890-0.933 | 0.920 | 0.899-0.940 |
|  | 61 – 75 | 0.901 | 0.868-0.932 | 0.950 | 0.923-0.976 | 0.968 | 0.939-0.984 |
| High | 18 – 30 | 0.777 | 0.753-0.800 | 0.764 | 0.734-0.795 | 0.766 | 0.731-0.799 |
|  | 31 – 45 | 0.818 | 0.798-0.836 | 0.788 | 0.763-0.813 | 0.791 | 0.763-0.816 |
|  | 46 – 60 | 0.875 | 0.853-0.897 | 0.880 | 0.849-0.909 | 0.886 | 0.853-0.917 |
|  | 61 – 75 | 0.929 | 0.905-0.950 | 0.934 | 0.901-0.961 | 0.929 | 0.890-0.961 |
| **Education & Age group (binary)** | | | |  |  |  |  |
| Low | 18 – 45 | 0.796 | 0.758-0.833 | 0.781 | 0.733-0.827 | 0.786 | 0.735-0.835 |
|  | 46 – 75 | 0.895 | 0.865-0.921 | 0.917 | 0.884-0.947 | 0.933 | 0.898-0.969 |
| Middle | 18 – 45 | 0.822 | 0.805-0.839 | 0.825 | 0.805-0.846 | 0.831 | 0.810-0.853 |
|  | 46 – 75 | 0.904 | 0.888-0.919 | 0.929 | 0.914-0.945 | 0.940 | 0.925-0.954 |
| High | 18 – 45 | 0.804 | 0.789-0.819 | 0.781 | 0.7613-0.800 | 0.783 | 0.762-0.804 |
|  | 46 – 75 | 0.898 | 0.881-0.914 | 0.903 | 0.880-0.924 | 0.905 | 0.881-0.928 |
| **Singular chronic condition & Age group ^b^** | | | | | | | |
| Anxiety or depression | 18 – 30 | 0.730 | 0.680-0.785 | 0.730 | 0.672-0.779 | 0.732 | 0.668-0.786 |
|  | 31 – 45 | 0.761 | 0.712-0.815 | 0.762 | 0.706-0.821 | 0.773 | 0.713-0.835 |
|  | 46 – 60 | 0.746 | 0.681-0.820 | 0.781 | 0.706-0.864 | 0.778 | 0.696-0.864 |
|  | 61 – 75 | 0.816 | 0.665-0.910 | 0.888 | 0.709-0.975 | 0.960 | 0.832-0.978 |
| Any other | 18 – 30 | 0.744 | 0.706-0.783 | 0.707 | 0.661-0.751 | 0.705 | 0.656-0.754 |
|  | 31 – 45 | 0.766 | 0.731-0.801 | 0.741 | 0.692-0.787 | 0.750 | 0.702-0.799 |
|  | 46 – 60 | 0.819 | 0.779-0.861 | 0.836 | 0.776-0.888 | 0.850 | 0.793-0.905 |
|  | 61 – 75 | 0.905 | 0.850-0.948 | 0.903 | 0.636-0.976 | 0.873 | 0.558-0.971 |
| **Singular chronic condition & Age group (binary) ^b^** | | | | | | | |
| Anxiety or depression | 18 – 45 | 0.745 | 0.711-0.783 | 0.746 | 0.706-0.786 | 0.752 | 0.706-0.793 |
|  | 46 – 75 | 0.764 | 0.705-0.826 | 0.794 | 0.730-0.872 | 0.797 | 0.731-0.876 |
| Any other | 18 – 45 | 0.760 | 0.735-0.784 | 0.730 | 0.697-0.762 | 0.734 | 0.699-0.769 |
|  | 46 – 75 | 0.863 | 0.832-0.892 | 0.877 | 0.829-0.918 | 0.889 | 0.840-0.932 |
| **Education & Singular chronic condition ^b^** | | | | | | | |
| Low | Anxiety or depression | 0.774 | 0.684-0.869 | 0.782 | 0.672-0.881 | 0.788 | 0.669-0.903 |
|  | Any other | 0.801 | 0.730-0.866 | 0.772 | 0.678-0.856 | 0.776 | 0.659-0.872 |
| Middle | Anxiety or depression | 0.758 | 0.712-0.805 | 0.770 | 0.720-0.816 | 0.784 | 0.727-0.833 |
|  | Any other | 0.845 | 0.820-0.873 | 0.824 | 0.788-0.859 | 0.839 | 0.801-0.878 |
| High | Anxiety or depression | 0.744 | 0.700-0.791 | 0.751 | 0.703-0.805 | 0.747 | 0.691-0.804 |
|  | Any other | 0.813 | 0.788-0.836 | 0.803 | 0.766-0.835 | 0.800 | 0.760-0.835 |

AUROC = Area Under the Receiver Operating Characteristic curve; CI = Confidence Interval. 95% confidence intervals of the AUROC shown. Green colour indicates an AUROC of ≥0.9 (the darker the green, the higher the AUROC), yellow an AUROC of 0.8 ≤ - < 0.9, and red indicates an AUROC of 0.7 ≤ - < 0.8. a = Anxiety/Depression/Anxiety and Depression frequencies and percentage vs. not present (total N = 19,902 (100%)). b = same as a, but for a sample of the total population that has only one chronic condition (n = 5,892 (100%)).

Table S7A. Cross-tabulations of frequencies of problems on the A/D dimension with a cut-off ≥2 compared to anxiety symptoms on the GAD-7 (cut-off ≥8), by age group and by singular chronic condition.

| **Age group** | **Anxiety (GAD-7 ≥8)** | | **Anxiety/Depression dimension** | | **Total** |
| --- | --- | --- | --- | --- | --- |
|  |  |  | No problems | Any problems |  |
| 18-30 | Absent | n | 1,273 | 1,213 | 2,486 |
|  |  | % of Total | 32% | 30% | 62% |
|  | Present | n | 193 | 1,310 | 1,503 |
|  |  | % of Total | 5% | 33% | 38% |
|  | Total | n | 1,466 | 2,523 | 3,989 |
|  |  | % of Total | 37% | 63% | 100% |
| 31-45 | Absent | n | 2,370 | 1,853 | 4,223 |
|  |  | % of Total | 40% | 31% | 70% |
|  | Present | n | 267 | 1,517 | 1,784 |
|  |  | % of Total | 4% | 25% | 30% |
|  | Total | n | 2,637 | 3,370 | 6,007 |
|  |  | % of Total | 44% | 56% | 100% |
| 46-60 | Absent | n | 2,614 | 1,656 | 4,270 |
|  |  | % of Total | 49% | 31% | 81% |
|  | Present | n | 90 | 928 | 1,018 |
|  |  | % of Total | 2% | 18% | 19% |
|  | Total | n | 2,704 | 2,584 | 5,288 |
|  |  | % of Total | 51% | 49% | 100% |
| 61-75 | Absent | n | 3,009 | 1,190 | 4,199 |
|  |  | % of Total | 65% | 26% | 91% |
|  | Present | n | 32 | 387 | 419 |
|  |  | % of Total | 1% | 8% | 9% |
|  | Total | n | 3,041 | 1,577 | 4,618 |
|  |  | % of Total | 66% | 34% | 100% |
| Total | Absent | N | 9,266 | 5,912 | 15,178 |
|  |  | % of Total | 47% | 30% | 76% |
|  | Present | N | 582 | 4,142 | 4,724 |
|  |  | % of Total | 3% | 21% | 24% |
|  | Total | N | 9,848 | 10,054 | 19,902 |
|  |  | % of Total | 50% | 51% | 100% |
| **Singular chronic condition** | **Anxiety (GAD-7 ≥8)** | | **Anxiety/Depression dimension** | | |
|  |  |  | No problems | Any problems | Total |
| Anxiety or depression | Absent | n | 56 | 546 | 602 |
|  |  | % of Total | 5% | 44% | 48% |
|  | Present | n | 17 | 629 | 646 |
|  |  | % of Total | 1% | 50% | 52% |
|  | Total | n | 73 | 1,175 | 1,248 |
|  |  | % of Total | 6% | 94% | 100% |
| Any other | Absent | n | 2,126 | 1,387 | 3,513 |
|  |  | % of Total | 46% | 30% | 76% |
|  | Present | n | 167 | 964 | 1,131 |
|  |  | % of Total | 4% | 21% | 24% |
|  | Total | n | 2,293 | 2,351 | 4,644 |
|  |  | % of Total | 49% | 51% | 100% |
| Total | Absent | n | 2,182 | 1,933 | 4,115 |
|  |  | % of Total | 37% | 33% | 70% |
|  | Present | n | 184 | 1,593 | 1,777 |
|  |  | % of Total | 3% | 27% | 30% |
|  | Total | n | 2,366 | 3,526 | 5,892 |
|  |  | % of Total | 40% | 60% | 100% |

Percentages are rounded to 0 decimal points. Red markings highlight the non-corresponding results.

Table S7B. Cross-tabulations of frequencies of problems on the A/D dimension with a cut-off ≥2 compared to depression symptoms on the PHQ-9 (cut-off ≥10), by age group and by singular chronic condition.

| **Age group** | **Depression (PHQ-9 ≥10)** | | **Anxiety/Depression dimension** | | **Total** |
| --- | --- | --- | --- | --- | --- |
|  |  |  | No problems | Any problems |  |
| 18-30 | Absent | n | 1,280 | 1,244 | 2,524 |
|  |  | % of Total | 32% | 31% | 63% |
|  | Present | n | 186 | 1,279 | 1,465 |
|  |  | % of Total | 5% | 32% | 37% |
|  | Total | n | 1,466 | 2,523 | 3,989 |
|  |  | % of Total | 37% | 63% | 100% |
| 31-45 | Absent | n | 2,432 | 2,018 | 4,450 |
|  |  | % of Total | 41% | 34% | 74% |
|  | Present | n | 205 | 1,352 | 1,557 |
|  |  | % of Total | 3% | 23% | 26% |
|  | Total | n | 2,637 | 3,370 | 6,007 |
|  |  | % of Total | 44% | 56% | 100% |
| 46-60 | Absent | n | 2,641 | 1,787 | 4,428 |
|  |  | % of Total | 50% | 34% | 84% |
|  | Present | n | 63 | 797 | 860 |
|  |  | % of Total | 1% | 15% | 16% |
|  | Total | n | 2,704 | 2,584 | 5,288 |
|  |  | % of Total | 51% | 49% | 100% |
| 61-75 | Absent | n | 3,012 | 1,267 | 4,279 |
|  |  | % of Total | 65% | 27% | 93% |
|  | Present | n | 29 | 310 | 339 |
|  |  | % of Total | 1% | 7% | 7% |
|  | Total | n | 3,041 | 1,577 | 4,618 |
|  |  | % of Total | 66% | 34% | 100% |
| Total | Absent | N | 9,365 | 6,316 | 15,681 |
|  |  | % of Total | 47% | 32% | 79% |
|  | Present | N | 483 | 3,738 | 4,221 |
|  |  | % of Total | 2% | 19% | 21% |
|  | Total | N | 9,848 | 10,054 | 19,902 |
|  |  | % of Total | 50% | 51% | 100% |
| **Singular chronic condition** | **Depression (PHQ-9 ≥10)** | | **Anxiety/Depression dimension** | | **Total** |
|  |  |  | No problems | Any problems |  |
| Anxiety or depression | Absent | n | 54 | 600 | 654 |
|  |  | % of Total | 4% | 48% | 52% |
|  | Present | n | 19 | 575 | 594 |
|  |  | % of Total | 2% | 46% | 48% |
|  | Total | n | 73 | 1,175 | 1,248 |
|  |  | % of Total | 6% | 94% | 100% |
| Any other | Absent | n | 2,144 | 1,478 | 3,622 |
|  |  | % of Total | 46% | 32% | 78% |
|  | Present | n | 149 | 873 | 1,022 |
|  |  | % of Total | 3% | 19% | 22% |
|  | Total | n | 2,293 | 2,351 | 4,644 |
|  |  | % of Total | 49% | 51% | 100% |
| Total | Absent | n | 2,198 | 2,078 | 4,276 |
|  |  | % of Total | 37% | 35% | 73% |
|  | Present | n | 168 | 1,448 | 1,616 |
|  |  | % of Total | 3% | 25% | 27% |
|  | Total | n | 2,366 | 3,526 | 5,892 |
|  |  | % of Total | 40% | 60% | 100% |

Percentages are rounded to 0 decimal points. Red markings highlight the non-corresponding results.

Table S7C. Cross-tabulations of frequencies of problems on the A/D dimension with a cut-off ≥2 compared to anxiety and depression symptoms on the GAD-7 (cut-off ≥8) and PHQ-9 (cut-off ≥10), by age group and by singular chronic condition.

| **Age group** | **Co-morbid anxiety & depression (GAD-7 ≥8 & PHQ-9 ≥10)** | | **Anxiety/Depression dimension** | | **Total** |
| --- | --- | --- | --- | --- | --- |
|  |  |  | No problems | Any problems |  |
| 18-30 | Absent | n | 1,334 | 1,470 | 2,804 |
|  |  | % of Total | 33% | 37% | 70% |
|  | Present | n | 132 | 1,053 | 1,185 |
|  |  | % of Total | 3% | 26% | 30% |
|  | Total | n | 1,466 | 2,523 | 3,989 |
|  |  | % of Total | 37% | 63% | 100% |
| 31-45 | Absent | n | 2,477 | 2,252 | 4,729 |
|  |  | % of Total | 41% | 38% | 79% |
|  | Present | n | 160 | 1,118 | 1,278 |
|  |  | % of Total | 3% | 19% | 21% |
|  | Total | n | 2,637 | 3,370 | 6,007 |
|  |  | % of Total | 44% | 56% | 100% |
| 46-60 | Absent | n | 2,669 | 1,958 | 4,627 |
|  |  | % of Total | 51% | 37% | 88% |
|  | Present | n | 35 | 626 | 661 |
|  |  | % of Total | 1% | 12% | 13% |
|  | Total | n | 2,704 | 2,584 | 5,288 |
|  |  | % of Total | 51% | 49% | 100% |
| 61-75 | Absent | n | 3,030 | 1,349 | 4,379 |
|  |  | % of Total | 66% | 29% | 95% |
|  | Present | n | 11 | 228 | 239 |
|  |  | % of Total | 0% | 5% | 5% |
|  | Total | n | 3,041 | 1,577 | 4,618 |
|  |  | % of Total | 66% | 34% | 100% |
| Total | Absent | N | 9,510 | 7,029 | 16,539 |
|  |  | % of Total | 47.8% | 35.3% | 83.1% |
|  | Present | N | 338 | 3,025 | 3,363 |
|  |  | % of Total | 1.7% | 15.2% | 16.9% |
|  | Total | N | 9,848 | 10,054 | 19,902 |
|  |  | % of Total | 49.5% | 50.5% | 100.0% |
| **Singular chronic condition** | **Co-morbid anxiety & depression (GAD-7 ≥8 & PHQ-9 ≥10)** | | **Anxiety/Depression dimension** | | **Total** |
|  |  |  | No problems | Any problems |  |
| Anxiety or depression | Absent | n | 62 | 696 | 758 |
|  |  | % of Total | 5% | 56% | 61% |
|  | Present | n | 11 | 479 | 490 |
|  |  | % of Total | 1% | 38% | 39% |
|  | Total | n | 73 | 1,175 | 1,248 |
|  |  | % of Total | 6% | 94% | 100% |
| Any other | Absent | n | 2,185 | 1,639 | 3,824 |
|  |  | % of Total | 47% | 35% | 82% |
|  | Present | n | 108 | 712 | 820 |
|  |  | % of Total | 2% | 15% | 18% |
|  | Total | n | 2,293 | 2,351 | 4,644 |
|  |  | % of Total | 49% | 51% | 100% |
| Total | Absent | n | 2,247 | 2,335 | 4,582 |
|  |  | % of Total | 38% | 40% | 78% |
|  | Present | n | 119 | 1,191 | 1,310 |
|  |  | % of Total | 2% | 20% | 22% |
|  | Total | n | 2,366 | 3,526 | 5,892 |
|  |  | % of Total | 40% | 60% | 100% |

Percentages are rounded to 0 decimal points. Red markings highlight the non-corresponding results.

Table S8A. Cross-tabulations of frequencies of problems on the A/D dimension with a cut-off ≥3 compared to anxiety symptoms on the GAD-7 (cut-off ≥8), by age group and by singular chronic condition.

| **Age group** | **Anxiety (GAD-7 ≥8)** | | **Anxiety/Depression dimension** | | **Total** |
| --- | --- | --- | --- | --- | --- |
|  |  |  | No problems | Any problems |  |
| 18-30 | Absent | n | 2,142 | 344 | 2,486 |
|  |  | % of Total | 54% | 9% | 62% |
|  | Present | n | 633 | 870 | 1,503 |
|  |  | % of Total | 16% | 22% | 38% |
|  | Total | n | 2,775 | 1,214 | 3,989 |
|  |  | % of Total | 70% | 30% | 100% |
| 31-45 | Absent | n | 3,755 | 468 | 4,223 |
|  |  | % of Total | 63% | 8% | 70% |
|  | Present | n | 771 | 1,013 | 1,784 |
|  |  | % of Total | 13% | 17% | 30% |
|  | Total | n | 4,526 | 1,481 | 6,007 |
|  |  | % of Total | 75% | 25% | 100% |
| 46-60 | Absent | n | 3,887 | 383 | 4,270 |
|  |  | % of Total | 74% | 7% | 81% |
|  | Present | n | 406 | 612 | 1,018 |
|  |  | % of Total | 8% | 12% | 19% |
|  | Total | n | 4,293 | 995 | 5,288 |
|  |  | % of Total | 81% | 19% | 100% |
| 61-75 | Absent | n | 4,012 | 187 | 4,199 |
|  |  | % of Total | 87% | 4% | 91% |
|  | Present | n | 184 | 235 | 419 |
|  |  | % of Total | 4% | 5% | 9% |
|  | Total | n | 4,196 | 422 | 4,618 |
|  |  | % of Total | 91% | 9% | 100% |
| Total | Absent | N | 13,796 | 1,382 | 15,178 |
|  |  | % of Total | 69% | 7% | 76% |
|  | Present | N | 1,994 | 2,730 | 4,724 |
|  |  | % of Total | 10% | 14% | 24% |
|  | Total | N | 15,790 | 4,112 | 19,902 |
|  |  | % of Total | 79% | 21% | 100% |
| **Singular chronic condition** | **Anxiety (GAD-7 ≥8)** | | **Anxiety/Depression dimension** | | **Total** |
|  |  |  | No problems | Any problems |  |
| Anxiety or depression | Absent | n | 327 | 275 | 602 |
|  |  | % of Total | 26% | 22% | 48% |
|  | Present | n | 131 | 515 | 646 |
|  |  | % of Total | 11% | 41% | 52% |
|  | Total | n | 458 | 790 | 1,248 |
|  |  | % of Total | 37% | 63% | 100% |
| Any other | Absent | n | 3,261 | 252 | 3,513 |
|  |  | % of Total | 70% | 5% | 76% |
|  | Present | n | 556 | 575 | 1,131 |
|  |  | % of Total | 12% | 12% | 24% |
|  | Total | n | 3,817 | 827 | 4,644 |
|  |  | % of Total | 82% | 18% | 100% |
| Total | Absent | n | 3,588 | 527 | 4,115 |
|  |  | % of Total | 61% | 9% | 70% |
|  | Present | n | 687 | 1,090 | 1,777 |
|  |  | % of Total | 12% | 19% | 30% |
|  | Total | n | 4,275 | 1,617 | 5,892 |
|  |  | % of Total | 73% | 27% | 100% |

Percentages are rounded to 1 decimal point. Red markings highlight the non-corresponding results.

Table S8B. Cross-tabulations of frequencies of problems on the A/D dimension with a cut-off ≥3 compared to depression symptoms on the PHQ-9 (cut-off ≥10), by age group and by singular chronic condition.

| **Age group** | **Depression (PHQ-9 ≥10)** | | **Anxiety/Depression dimension** | | **Total** |
| --- | --- | --- | --- | --- | --- |
|  |  |  | No problems | Any problems |  |
| 18-30 | Absent | n | 2,145 | 379 | 2,524 |
|  |  | % of Total | 54% | 10% | 63% |
|  | Present | n | 630 | 835 | 1,465 |
|  |  | % of Total | 16% | 21% | 37% |
|  | Total | n | 2,775 | 1,214 | 3,989 |
|  |  | % of Total | 70% | 30% | 100% |
| 31-45 | Absent | n | 3,888 | 562 | 4,450 |
|  |  | % of Total | 65% | 9% | 74% |
|  | Present | n | 638 | 919 | 1,557 |
|  |  | % of Total | 11% | 15% | 26% |
|  | Total | n | 4,526 | 1,481 | 6,007 |
|  |  | % of Total | 75% | 25% | 100% |
| 46-60 | Absent | n | 4,017 | 411 | 4,428 |
|  |  | % of Total | 76% | 8% | 84% |
|  | Present | n | 276 | 584 | 860 |
|  |  | % of Total | 5% | 11% | 16% |
|  | Total | n | 4,293 | 995 | 5,288 |
|  |  | % of Total | 81% | 19% | 100% |
| 61-75 | Absent | n | 4,060 | 219 | 4,279 |
|  |  | % of Total | 88% | 5% | 93% |
|  | Present | n | 136 | 203 | 339 |
|  |  | % of Total | 3% | 4% | 7% |
|  | Total | n | 4,196 | 422 | 4,618 |
|  |  | % of Total | 91% | 9% | 100% |
| Total | Absent | N | 14,110 | 1,571 | 15,681 |
|  |  | % of Total | 71% | 8% | 79% |
|  | Present | N | 1,680 | 2,541 | 4,221 |
|  |  | % of Total | 8% | 13% | 21% |
|  | Total | N | 15,790 | 4,112 | 19,902 |
|  |  | % of Total | 79% | 21% | 100% |
| **Singular chronic condition** | **Depression (PHQ-9 ≥10)** | | **Anxiety/Depression dimension** | | **Total** |
|  |  |  | No problems | Any problems |  |
| Anxiety or depression | Absent | n | 334 | 320 | 654 |
|  |  | % of Total | 27% | 26% | 52% |
|  | Present | n | 124 | 470 | 594 |
|  |  | % of Total | 10% | 38% | 48% |
|  | Total | n | 458 | 790 | 1,248 |
|  |  | % of Total | 37% | 63% | 100% |
| Any other | Absent | n | 3,326 | 296 | 3,622 |
|  |  | % of Total | 72% | 6% | 78% |
|  | Present | n | 491 | 531 | 1,022 |
|  |  | % of Total | 11% | 11% | 22% |
|  | Total | n | 3,817 | 827 | 4,644 |
|  |  | % of Total | 82% | 18% | 100% |
| Total | Absent | n | 3,660 | 616 | 4,276 |
|  |  | % of Total | 62% | 11% | 73% |
|  | Present | n | 615 | 1,001 | 1,616 |
|  |  | % of Total | 10% | 17% | 27% |
|  | Total | n | 4,275 | 1,617 | 5,892 |
|  |  | % of Total | 73% | 27% | 100% |

Percentages are rounded to 0 decimal points. Red markings highlight the non-corresponding results.

Table S8C. Cross-tabulations of frequencies of problems on the A/D dimension with a cut-off ≥3 compared to anxiety and depression symptoms on the GAD-7 (cut-off ≥8) and PHQ-9 (cut-off ≥10), by age group and by singular chronic condition.

| **Age group** | **Co-morbid anxiety & depression (GAD-7 ≥8 & PHQ-9 ≥10)** | | **Anxiety/Depression dimension** | | **Total** |
| --- | --- | --- | --- | --- | --- |
|  |  |  | No problems | Any problems |  |
| 18-30 | Absent | n | 2,322 | 482 | 2,804 |
|  |  | % of Total | 58% | 12% | 70% |
|  | Present | n | 453 | 732 | 1,185 |
|  |  | % of Total | 11% | 18% | 30% |
|  | Total | n | 2,775 | 1,214 | 3,989 |
|  |  | % of Total | 70% | 30% | 100% |
| 31-45 | Absent | n | 4,056 | 673 | 4,729 |
|  |  | % of Total | 68% | 11% | 79% |
|  | Present | n | 470 | 808 | 1,278 |
|  |  | % of Total | 8% | 14% | 21% |
|  | Total | n | 4,526 | 1,481 | 6,007 |
|  |  | % of Total | 75% | 25% | 100% |
| 46-60 | Absent | n | 4,118 | 509 | 4,627 |
|  |  | % of Total | 78% | 10% | 88% |
|  | Present | n | 175 | 486 | 661 |
|  |  | % of Total | 3% | 9% | 13% |
|  | Total | n | 4,293 | 995 | 5,288 |
|  |  | % of Total | 81% | 19% | 100% |
| 61-75 | Absent | n | 4,124 | 255 | 4,379 |
|  |  | % of Total | 89% | 6% | 95% |
|  | Present | n | 72 | 167 | 239 |
|  |  | % of Total | 2% | 4% | 5% |
|  | Total | n | 4,196 | 422 | 4,618 |
|  |  | % of Total | 91% | 9% | 100% |
| Total | Absent | N | 14,620 | 1,919 | 16,539 |
|  |  | % of Total | 74% | 10% | 83% |
|  | Present | N | 1,170 | 2,193 | 3,363 |
|  |  | % of Total | 6% | 11% | 17% |
|  | Total | N | 15,790 | 4,112 | 19,902 |
|  |  | % of Total | 79% | 21% | 100% |
| **Singular chronic condition** | **Co-morbid anxiety & depression (GAD-7 ≥8 & PHQ-9 ≥10)** | | **Anxiety/Depression dimension** | | **Total** |
|  |  |  | No problems | Any problems |  |
| Anxiety or depression | Absent | n | 369 | 389 | 758 |
|  |  | % of Total | 30% | 31% | 61% |
|  | Present | n | 89 | 401 | 490 |
|  |  | % of Total | 7% | 32% | 39% |
|  | Total | n | 458 | 790 | 1,248 |
|  |  | % of Total | 37% | 63% | 100% |
| Any other | Absent | n | 3,463 | 361 | 3,824 |
|  |  | % of Total | 75% | 8% | 82% |
|  | Present | n | 354 | 466 | 820 |
|  |  | % of Total | 8% | 10% | 18% |
|  | Total | n | 3,817 | 827 | 4,644 |
|  |  | % of Total | 82% | 18% | 100% |
| Total | Absent | n | 3,832 | 750 | 4,582 |
|  |  | % of Total | 65% | 13% | 78% |
|  | Present | n | 443 | 867 | 1,310 |
|  |  | % of Total | 8% | 15% | 22% |
|  | Total | n | 4,275 | 1,617 | 5,892 |
|  |  | % of Total | 73% | 27% | 100% |

Percentages are rounded to 1 decimal point. Red markings highlight the non-corresponding results.

Table S9. Sensitivity, specificity, PPV, NPV and accuracy of the A/D dimension compared to the diagnostic groups (GAD-7, PHQ-9 and co-morbid GAD-7 and PHQ-9) for the total population, by age group and by singular chronic condition using a cut-off score of ≥2 on the A/D dimension.

|  | | Sensitivity | Specificity | PPV | NPV | Accuracy | Youden’s Index |
| --- | --- | --- | --- | --- | --- | --- | --- |
| Anxiety present (GAD-7 ≥8) | |  |  |  |  |  |  |
| Total sample | | 88% | 61% | 41% | 94% | 67% | 0.49 |
| Age category | 18-30 | 87% | 51% | 52% | 87% | 65% | 0.38 |
|  | 31-45 | 85% | 56% | 45% | 90% | 65% | 0.41 |
|  | 46-60 | 91% | 61% | 36% | 97% | 67% | 0.52 |
|  | 61-75 | 92% | 72% | 25% | 99% | 74% | 0.64 |
| Singular chronic condition | Anxiety or depression | 97% | 9% | 54% | 77% | 55% | 0.07 |
|  | Any other | 85% | 61% | 41% | 93% | 67% | 0.46 |
| Depression present (PHQ-9 ≥10) | | | | | | |  |
| Total sample | | 89% | 60% | 37% | 95% | 66% | 0.48 |
| Age category | 18-30 | 87% | 51% | 51% | 87% | 64% | 0.38 |
|  | 31-45 | 87% | 55% | 40% | 92% | 63% | 0.41 |
|  | 46-60 | 93% | 60% | 31% | 98% | 65% | 0.52 |
|  | 61-75 | 91% | 70% | 20% | 99% | 72% | 0.62 |
| Singular chronic condition | Anxiety or depression | 97% | 8% | 49% | 74% | 50% | 0.05 |
|  | Any other | 85% | 59% | 37% | 94% | 65% | 0.45 |
| Co-morbid anxiety & depression present (GAD-7 ≥8 & PHQ-9 ≥10) | | | | | | |  |
| Total sample | | 90% | 58% | 30% | 97% | 63% | 0.48 |
| Age category | 18-30 | 89% | 48% | 42% | 91% | 60% | 0.36 |
|  | 31-45 | 87% | 52% | 33% | 94% | 60% | 0.40 |
|  | 46-60 | 95% | 58% | 24% | 99% | 62% | 0.52 |
|  | 61-75 | 95% | 69% | 14% | 100% | 71% | 0.65 |
| Singular chronic condition | Anxiety or depression | 98% | 8% | 41% | 85% | 43% | 0.06 |
|  | Any other | 87% | 57% | 30% | 95% | 62% | 0.44 |

PPV = positive predictive value; NPV = negative predictive value. Percentages are rounded to 0 decimal points.

Table S10. Sensitivity, specificity, PPV, NPV and accuracy of the A/D dimension compared to the diagnostic groups (GAD-7, PHQ-9 and co-morbid GAD-7 and PHQ-9) for the total population, by age group and by singular chronic condition, using a cut-off score of ≥3 on the A/D dimension.

|  | | Sensitivity | Specificity | PPV | NPV | Accuracy | Youden’s Index |
| --- | --- | --- | --- | --- | --- | --- | --- |
| Anxiety present (GAD-7 ≥8) | |  |  |  |  |  |  |
| Total sample | | 58% | 91% | 66% | 87% | 83% | 0.49 |
| Age category | 18-30 | 58% | 86% | 72% | 77% | 76% | 0.44 |
|  | 31-45 | 57% | 89% | 68% | 83% | 79% | 0.46 |
|  | 46-60 | 60% | 91% | 62% | 91% | 85% | 0.51 |
|  | 61-75 | 56% | 96% | 56% | 96% | 92% | 0.52 |
| Singular chronic condition | Anxiety or depression | 80% | 51% | 65% | 71% | 67% | 0.31 |
|  | Any other | 54% | 93% | 70% | 85% | 83% | 0.47 |
| Depression present (PHQ-9 ≥10) | | | | | | |  |
| Total sample | | 60% | 90% | 62% | 89% | 84% | 0.50 |
| Age category | 18-30 | 57% | 85% | 69% | 77% | 75% | 0.42 |
|  | 31-45 | 59% | 87% | 62% | 86% | 80% | 0.46 |
|  | 46-60 | 68% | 91% | 59% | 94% | 87% | 0.59 |
|  | 61-75 | 60% | 95% | 48% | 97% | 92% | 0.55 |
| Singular chronic condition | Anxiety or depression | 79% | 51% | 59% | 73% | 64% | 0.30 |
|  | Any other | 52% | 92% | 64% | 87% | 83% | 0.44 |
| Co-morbid anxiety & depression present (GAD-7 ≥8 & PHQ-9 ≥10) | | | | | | |  |
| Total sample | | 65% | 88% | 53% | 93% | 85% | 0.54 |
| Age category | 18-30 | 62% | 83% | 60% | 84% | 77% | 0.45 |
|  | 31-45 | 63% | 86% | 55% | 90% | 81% | 0.49 |
|  | 46-60 | 74% | 89% | 49% | 96% | 87% | 0.63 |
|  | 61-75 | 70% | 94% | 40% | 98% | 93% | 0.64 |
| Singular chronic condition | Anxiety or depression | 82% | 49% | 51% | 81% | 62% | 0.31 |
|  | Any other | 57% | 91% | 56% | 91% | 85% | 0.47 |

PPV = positive predictive value; NPV = negative predictive value. Percentages are rounded to 0 decimal points.

Table S11. Sensitivity, specificity, PPV, NPV and accuracy of the A/D dimension compared to the diagnostic groups (GAD-7, PHQ-9 and co-morbid GAD-7 and PHQ-9) for the total population, by age group and by singular chronic condition using a cut-off score of ≥4 on the A/D dimension.

|  | | Sensitivity | Specificity | PPV | NPV | Accuracy | Youden’s Index |
| --- | --- | --- | --- | --- | --- | --- | --- |
| Anxiety present (GAD-7 ≥8) | |  |  |  |  |  |  |
| Total sample | | 25% | 99% | 84% | 81% | 81% | 0.24 |
| Age category | 18-30 | 26% | 97% | 82% | 68% | 70% | 0.23 |
|  | 31-45 | 26% | 98% | 86% | 76% | 77% | 0.24 |
|  | 46-60 | 26% | 99% | 83% | 85% | 85% | 0.25 |
|  | 61-75 | 19% | 100% | 86% | 92% | 92% | 0.18 |
| Singular chronic condition | Anxiety or depression | 42% | 91% | 84% | 60% | 66% | 0.33 |
|  | Any other | 18% | 99% | 87% | 79% | 79% | 0.17 |
| Depression present (PHQ-9 ≥10) | | | | | | |  |
| Total sample | | 28% | 98% | 81% | 83% | 83% | 0.26 |
| Age category | 18-30 | 27% | 97% | 82% | 69% | 71% | 0.23 |
|  | 31-45 | 28% | 98% | 81% | 79% | 80% | 0.26 |
|  | 46-60 | 30% | 99% | 81% | 88% | 88% | 0.29 |
|  | 61-75 | 21% | 100% | 78% | 94% | 94% | 0.20 |
| Singular chronic condition | Anxiety or depression | 43% | 89% | 78% | 63% | 67% | 0.32 |
|  | Any other | 19% | 99% | 83% | 81% | 81% | 0.18 |
| Co-morbid anxiety & depression present (GAD-7 ≥8 & PHQ-9 ≥10) | | | | | | |  |
| Total sample | | 32% | 98% | 75% | 88% | 87% | 0.29 |
| Age category | 18-30 | 30% | 96% | 74% | 76% | 76% | 0.25 |
|  | 31-45 | 32% | 97% | 76% | 84% | 83% | 0.29 |
|  | 46-60 | 36% | 98% | 74% | 92% | 90% | 0.35 |
|  | 61-75 | 27% | 99% | 71% | 96% | 96% | 0.27 |
| Singular chronic condition | Anxiety or depression | 49% | 88% | 73% | 73% | 73% | 0.37 |
|  | Any other | 22% | 99% | 76% | 85% | 85% | 0.20 |

PPV = positive predictive value; NPV = negative predictive value. Percentages are rounded to 0 decimal points.

Table S12. Sensitivity, specificity, PPV, NPV and accuracy of the A/D dimension compared to the diagnostic groups (GAD-7, PHQ-9 and co-morbid GAD-7 and PHQ-9) for the total population, by age group and by singular chronic condition using a cut-off score of 5 on the A/D dimension.

|  | | Sensitivity | Specificity | PPV | NPV | Accuracy | Youden’s Index |
| --- | --- | --- | --- | --- | --- | --- | --- |
| Anxiety present (GAD-7 ≥8) | |  |  |  |  |  |  |
| Total sample | | 9% | 100% | 88% | 78% | 78% | 0.08 |
| Age category | 18-30 | 9% | 99% | 84% | 64% | 65% | 0.08 |
|  | 31-45 | 9% | 99% | 88% | 72% | 73% | 0.09 |
|  | 46-60 | 8% | 100% | 91% | 82% | 82% | 0.08 |
|  | 61-75 | 6% | 100% | 93% | 91% | 91% | 0.06 |
| Singular chronic condition | Anxiety or depression | 16% | 98% | 91% | 52% | 56% | 0.14 |
|  | Any other | 5% | 100% | 88% | 77% | 77% | 0.05 |
| Depression present (PHQ-9 ≥10) | | | | | | |  |
| Total sample | | 9% | 100% | 85% | 80% | 80% | 0.09 |
| Age category | 18-30 | 9% | 99% | 83% | 65% | 66% | 0.08 |
|  | 31-45 | 10% | 99% | 86% | 76% | 76% | 0.10 |
|  | 46-60 | 10% | 100% | 88% | 85% | 85% | 0.09 |
|  | 61-75 | 7% | 100% | 86% | 93% | 93% | 0.07 |
| Singular chronic condition | Anxiety or depression | 17% | 98% | 88% | 56% | 59% | 0.15 |
|  | Any other | 6% | 100% | 87% | 79% | 79% | 0.06 |
| Co-morbid anxiety & depression present (GAD-7 ≥8 & PHQ-9 ≥10) | | | | | | |  |
| Total sample | | 11% | 99% | 81% | 85% | 85% | 0.11 |
| Age category | 18-30 | 10% | 99% | 76% | 72% | 72% | 0.09 |
|  | 31-45 | 12% | 99% | 82% | 81% | 81% | 0.11 |
|  | 46-60 | 12% | 100% | 85% | 89% | 89% | 0.12 |
|  | 61-75 | 10% | 100% | 86% | 95% | 95% | 0.10 |
| Singular chronic condition | Anxiety or depression | 20% | 98% | 86% | 65% | 67% | 0.17 |
|  | Any other | 7% | 100% | 79% | 83% | 83% | 0.06 |

PPV = positive predictive value; NPV = negative predictive value. Percentages are rounded to 0 decimal points.
